# Unveiling Heterospecific Pollen Deposition in *Ranunculus* Plants Along a Land‐Use Gradient Through DNA Metabarcoding

**DOI:** 10.1002/ece3.71184

**Published:** 2025-03-27

**Authors:** Susanne Werle, Anna Preußner, Kenneth Kuba, Sara Diana Leonhardt, Alexander Keller

**Affiliations:** ^1^ Plant‐Insect Interactions, TUM School of Life Science Systems Technical University of Munich (TUM) Freising Germany; ^2^ Cellular and Organismic Networks, Faculty of Biology LMU Munich Munich Germany

**Keywords:** biodiversity exploratories, cross‐pollination, heterospecific pollen, molecular ecology, plant diversity, pollen DNA metabarcoding, pollinator decline

## Abstract

Animal pollination, the transfer of pollen by animal agents, is essential for plant reproduction. Methods like microscopy and DNA metabarcoding have been used to investigate pollen transport and plant–pollinator interactions. DNA metabarcoding, in particular, is a reliable method to identify the origins of mixed pollen samples. Although it has mainly been used to study pollinators' dietary patterns, it does not provide insights from the plant's perspective, such as the type of viable pollen received. We aimed to explore the potential of DNA metabarcoding to analyse heterospecific pollen transfer to plants in semi‐natural and agricultural landscapes along a land‐use intensity gradient. We collected stigmas of three closely related *Ranunculus* species ( *R. acris* , *R. bulbosus* and *R. repens* ) from 20 grassland plots in Germany with varying land‐use intensities and flowering plant diversity and subjected them to internal transcribed spacer 2 (ITS2) metabarcoding. Our results revealed a nonlinear relationship between flowering plant species richness and heterospecific pollen richness on *Ranunculus* stigmas. The lowest heterospecific pollen diversity occurred in landscapes with intermediate plant species richness, whereas plots with low or high richness showed greater heterospecific pollen diversity. Reduced plant species richness, found mostly on intermediate and high LUI plots, forces pollinators to visit multiple plant species and thus increases heterospecific pollen transfer. Plots with intermediate plant species richness, on the contrary, likely provide a balanced mix of resources for pollinators, visiting multiple plant species within a foraging round and thus decreasing the amount of heterospecific pollen. Increased heterospecific pollen at high‐richness plots may result from competition in pollinator‐rich communities. Our results show that DNA metabarcoding is a powerful tool for assessing heterospecific pollen diversity, revealing that pollen transfer is heavily influenced by plant community composition. This approach provides novel insights into pollinator fidelity and potential pollination outcomes across diverse environments.

## Graphical abstract

We used DNA metabarcoding to explore heterospecific pollen transfer to three *Ranunculus* species in semi‐natural and agricultural landscapes across a land‐use intensity gradient. Our results show that flowering plant diversity significantly influences the diversity of pollen deposited on plant stigmas, with intermediate plant species landscapes exhibiting the lowest pollen diversity. This study highlights the potential of DNA metabarcoding to assess pollen transfer dynamics and their ecological implications in varying landscapes.

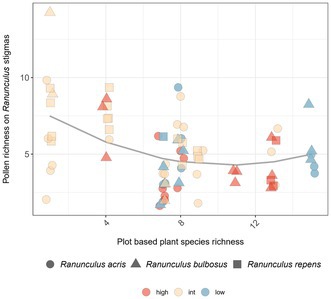

## 1 Introduction

The global diversity of plants and pollinators is vast (Abrol 2012) and leads to complex interaction networks. However, the current and continuous decrease in biodiversity is threatening species interactions and associated ecosystem functions, like animal pollination (Biesmeijer et al. 2006), crucial for nature and human society (Loreau et al. 2001). Gaining more insights into interaction structures and outcomes (here: pollination) has therefore become a high priority in ecological research. Most wild plants and agricultural crops are pollinated by insects, especially by wild bees (Potts et al. 2010). More specifically, around 87.5% of angiosperms are estimated to depend on animals for cross‐pollination and thus reproduction (Ollerton et al. 2011). Cross‐pollination refers to the transfer of pollen between different plant genotypes within the same species, facilitating gene flow and enhancing genetic diversity in both cultivated crops and wild plants (Chumacero de Schawe et al. 2013). However, it is important to note that animal pollinators do not always exclusively carry pollen from different genotypes of the same plant species; they may also transfer pollen within the same plant or between different species (heterospecific pollen transfer). This can influence the overall efficiency of cross‐pollination and affect reproductive success (Ramírez and Davenport 2013). Studies on several different crops have shown an increased yield and, in some crops, also an improved quality with cross‐pollination (Schneider et al. 2009; Klein et al. 2007; Dung et al. 2021).

Although it is clear that many plants need flower visitors for pollination, it is often unclear which animals actually serve as pollen vectors, how many pollinators are required for successful pollen transfer, or whether/how pollination success is affected by the surrounding landscape. In fact, many flower visitors are generalists and visit more than only a single plant species for pollen and nectar collection in a single foraging trip (Kriesell et al. 2017; Hicks et al. 2016). Such behaviour increases the chances for heterospecific pollen transfer, that is, that the plant individual receives pollen from a different plant species. Additionally, pollen from wind‐pollinated plants is ubiquitous and attaches to receptor stigmas with unknown consequences for the reproduction of heterospecific plants. In fact, heterospecific pollen and its composition can show detrimental effects for seed production (Arceo‐Gómez and Ashman 2011) via different mechanisms, for example, unsuccessful germination of closely related pollen or physical and chemical suppression of other pollens (Gardner and Macnair 2000; Murphy and Aarssen 1995; Harder et al. 1993). Moreover, the ongoing biodiversity crisis poses significant risks to interactions between plants and pollinators (Biesmeijer et al. 2006). Understanding the factors that shape plant–pollinator networks, particularly the dynamics of pollen transfer, is thus essential to mitigate these impacts. It would therefore be useful to have tools to measure heterospecific pollen diversity and to identify factors influencing the composition of deposited pollen.

Although many flowering plants rely on animal pollination for reproduction (Goulson 1999), the influence of landscape features and ecological conditions, such as land‐use intensification (LUI) and surrounding plant species diversity, on pollen transfer remains underexplored. This gap in knowledge is largely due to limitations of traditional approaches used to taxonomically identify pollen loads. For example, light microscopy has been applied in studies of plant–pollinator interactions to identify pollen loads on plant stigmas and pollinators (Briggs et al. 2016; Fang and Huang 2013; Beattie 1971; Erdtman 1966), but microscopy‐based methods require trained researchers and are time‐ and cost‐intensive (Smart et al. 2017). Furthermore, they often fall short in providing high taxonomic resolution, with identifications often limited to genus level or higher (Mullins and Emberlin 1997). To overcome these challenges, DNA metabarcoding has emerged as a powerful tool for identifying plant species in complex pollen samples containing diverse taxa (Keller et al. 2015; Lucas et al. 2018; Pornon et al. 2017). This method has been successfully used to study pollen collected by pollinators, offering insights into their foraging behaviour and preferences (Elliott et al. 2021; Lucas et al. 2018; Pornon et al. 2017). It has, however, not yet been applied to analyse stigma‐deposited pollen. Such a method might, however, prove useful to trace heterospecific pollen deposition and fill gaps in the knowledge of pollination ecology, providing insights into pollen flow and pollination mechanisms in natural and human‐modified landscapes.

Pollinator diversity typically increases with plant species richness (Weiner et al. 2011; Peters et al. 2022), and the frequency of pollinator visits positively affects the number of flowering plant species (Ebeling et al. 2008). In landscapes with low LUI and high plant species richness, the greater diversity of flowering plants and pollinators may lead to more stochastic interactions, potentially increasing the likelihood of heterospecific pollen deposition. Alternatively, a more diverse plant community may facilitate stronger niche separation among pollinators and foster modularity within the pollination network, thereby reducing heterospecific pollen transfer (Morales and Traveset 2008). These dynamics highlight a potential trade‐off between increased stochastic chance for cross‐species pollen transfer in diverse communities and the ecological pressures that drive niche differentiation and pollinator specialisation. In fact, the richness of pollinators visiting individual *Ranunculus* plants at the same field sites as studied in our study (Weiner et al. 2014) increased with the richness of surrounding plant species (see Figures S10–S12 and Table S5). Looking at pollinator richness on all flowering plants from the same study, we see an increase with increasing plant species richness, with a fast increase of pollinator richness on intermediate (yellow) and high (red) LUI plots and a slower pollinator richness slope in low LUI plots (blue) (see Figures S13 and S14; Table S6). A more recent dataset from 2020 and 2021 (Parreño et al. 2025) restricted only to bee species on *Ranunculus* flowers showed a similar pattern as seen in Figure S14, where bee species richness on *Ranunculus* flowers (*Bee richness on Ranunculus*) correlated positively with plot based plant species richness (*Average plant species richness*; see Figure S15, Table S7). Moreover, the two most abundant Megachilid solitary bee species, *Osmia cornuta* and *Osmia bicornis* , were shown to forage primarily on Ranunculaceae and Rosaceae on high LUI plots, while they foraged on pollen from a wider variety of plant species on low LUI plots with higher plant diversity (Peters et al. 2022). The consequences of this variation in pollinator visitation patterns for heterospecific pollen deposition remain, however, unknown and are addressed in this study.

We applied DNA metabarcoding to analyse pollen deposited on the stigmas of three *Ranunculus* species to examine heterospecific pollen deposition along a LUI and plant diversity gradient and gain deeper insight into how ecological factors shape pollen deposition dynamics. We hypothesised that the diversity of heterospecific pollen on *Ranunculus* stigmas is influenced by the surrounding plant species richness and land‐use intensity. Specifically, we investigated two alternative hypotheses, that is, whether (1) greater plant species richness increases heterospecific pollen deposition due to the higher diversity of surrounding flowering plants, (2) greater plant species richness reduces heterospecific pollen deposition by encouraging stronger niche differentiation among pollinators.

## 2 Material and Methods

### 2.1 Sample Collection and Study Design

Fieldwork was conducted in May 2022 on Swabian Alb (ALB) and Hanich‐Dün (HAI) plots within the research framework of the Biodiversity Exploratories (www.biodiversity‐exploratories.de; Fischer et al. 2010). They consist of various grassland plots with a broad variation of typical land‐use management forms in Germany. The intensity of these several types of land use is described quantitatively by the land‐use intensity index (LUI). It combines fertilisation and livestock grazing intensity as well as mowing frequency (Blüthgen et al. 2012) and allows for studies along a LUI gradient to reveal effects of different land‐use intensities and parameters on numerous aspects of biodiversity. Samples were collected on 20 experimental grassland plots (50 × 50 m) with either high, intermediate or low land‐use intensity. Detailed information on the plots is given by Fischer et al. (2010). The LUI was calculated as a global mean of grassland management for the regions ALB and HAI (overall) for the year of 2021 according to Blüthgen et al. (2012), based on information from the land owners on mowing, grazing and fertilisation (Vogt et al. 2019) using the LUI calculation tool (Andreas Ostrowski et al. 2020) implemented in BExIS ((re3data.org 2016) http://doi.org/10.17616/R32P9Q).

Sampling was conducted during the first week of May 2022 (02.05.2022–6.05.2022) in the Swabian Alb on three low LUI plots (AEG 03, AEG 07, AEG 10), three intermediate LUI plots (AEG 04, AEG 06, AEG 17) and three high LUI plots (AEG 02, AEG 21, AEG 46). Towards the end of May (23.05.2022–25.05.2022), samples were collected in Hainich‐Dün on three low LUI plots (HEG 17, HEG 19, HEG 42), five intermediate LUI plots (HEG 03, HEG 04, HEG 05, HEG 06, HEG 08) and three high LUI plots (HEG 02, HEG 07, HEG 12). Sampling plots were categorised into low, intermediate and high LUI plots based on their overall LUI value for 2021, using percentile calculations: low (0–1.44), intermediate (1.45–2.13) and high (2.14–3.85). The exact location of all the sampled plots can be found in Figures S1–S3. Coordinates of all plots can be found in Table S1.

*Ranunculus* was chosen as our target group as this was the only genus present throughout the complete set of plots from low to high intensities. We selected three *Ranunculus* species, *R. acris* , *R. bulbosus* and *R. repens* , which are among the most important species for wild bees within the Ranunculaceae (Westrich 2019) and were found on most of the plots within the Biodiversity Exploratories. Most *Ranunculus* species are self‐incompatible, requiring cross‐pollination to ensure successful reproduction, as described by Kipling and Warren (2014) for *R. acris* and *R. repens* . Even though there is limited evidence for the hybridisation of *R. acris* , *R. repens* and *R. bulbosus* , hybridisation has been documented in Europe for aquatic *Ranunculus* species (Bobrov et al. 2022) and between *R. acris* and *R. uncinatus* in Alaska (Welsh 1974). Therefore, we assume that hybridisation can also be a possibility for our three *Ranunculus* species within this study. For each *Ranunculus* species, three patches per plot with a radius of 5 m were defined with a distance of at least 10 m to each other. Stigmas were picked with curved forceps and transferred into a falcon tube with spring steel forceps. We pooled all 16–20 stigmas of the same patch and multiple individuals in 15 mL falcon tubes. Pollen self‐contamination through the plant's anthers was avoided as much as possible by cleaning both forceps with 10% bleach and drying completely to remove any DNA residues before and after finishing a patch.

Vegetation assessments were made simultaneously to stigma sampling to quantify the plant diversity at the respective times. Flowering plant species richness was assessed per plot using ten 1 × 1 m quadrants and identifying all flowering plant species as well as the number of open flowers in each quadrant (https://doi.org/10.5281/zenodo.13145226 [Werle, Kuba, et al. 2024]). Positions for all ten quadrants on plots can be found in Figure S4. Not all three *Ranunculus* species could be found on every plot in both Exploratories within the sampling time frame. The exact sample numbers and distribution of samples collected on plots differing in LUI are illustrated in Figure S5, with 94 samples in total. In spring 2022, temperatures were lower than expected and flowers started blooming later, resulting in a lower sample size of *Ranunculus* stigmas for the Swabian Alb (*n* = 34) than for Hainich‐Dün (*n* = 60).

### 2.2 Sample Preparation

The collected stigmas were examined under a stereo microscope (Zeiss Stemi 305; Carl Zeiss AG, Oberkochen, Germany) to roughly assess the amount of pollen adherent to them. Most of the pollen was comparatively small and yellow, likely representing *Ranunculus* pollen from the flower's own anthers or other individuals. Therefore, only heterospecific pollen is assessed in the following. However, small and medium‐sized white pollen could also be found on most of the stigmas (Figure S6). Prior to DNA extraction, all stigmas were gently washed to detach the pollen. Stigmas from one falcon tube were transferred into two separate 2‐mL microcentrifuge tubes (tube A and B), which were then filled with 1× phosphate‐buffered saline (PBS; VWR Chemicals, Ohio, USA). The filled tubes were vortexed at 1300 rpm for 1 min only to avoid damaging stigmas and releasing undesired *Ranunculus* DNA from the host plant to the pollen sample. The washed stigmas without pollen were then removed from the tubes, and the remaining liquid was centrifuged at 13,000 rpm for 2 min. The supernatant of tube B as well as half of the supernatant of tube A for each sample was then discarded; the pellet of tube B was resuspended and transferred into tube A. Tube B was then washed with 200 μL 1× PBS to transfer remaining pollen to tube A as well. The washing process was repeated for all samples, which were then stored at −20°C.

### 2.3 DNA Extraction, PCR Amplification and MiSeq Sequencing

Prior to DNA extraction, samples were completely thawed and then centrifuged at 11,000 g for 10 min. Samples were extracted using the NucleoSpin 96 Food kit (Macherey‐Nagel GmbH & Co. KG, Düren, Germany) following the manufacturer's instructions with minor modifications as recommended for mixed pollen samples (Campos et al. 2021). The volume of Buffer CF was reduced to 400 μL as recommended in the vendor's support protocol for pollen extraction. Incubation time with Proteinase K was extended to 4 h at 65°C. Tungsten carbide beads were not utilised to disrupt the pollen, thus preventing additional host DNA from dispersing in the sample. For DNA metabarcoding, the ITS2 dual‐indexing and library preparation strategy specified by Sickel et al. (2016) was employed. Primer—and index sequences are provided in Tables S2 and S3. *Holcus lanatus* was chosen to generate 12 positive controls to monitor cross‐contamination during laboratory work. Additionally, 12 no‐library negative controls as described by Richardson (2022) were used to detect contamination that occurred during field and laboratory work. To index the stigma and control samples, the protocol outlined by Sickel et al. (2016) was followed, assigning a unique combination of forward and reverse primer sequences to each sample. To minimise laboratory contamination, the PCR was prepared under a PCR hood. The work area was cleaned thoroughly with 10% bleach and exposed to ultraviolet light for 15 min, along with the required pipettes and tips, before handling samples. To verify the success of each PCR and the approximate length of 490 base pairs, gel electrophoresis was performed using E‐Gel Double Combs 1% agarose with SYBR Safe (Thermo Fisher Scientific, Life Technologies, Carlsbad, CA, USA). Normalised PCR samples were pooled and then further purified with AMPure XP beads (Beckman Coulter GmbH, Krefeld, Germany) by adding an equivalent amount of beads to 0.8 of the total pool volume. After vortexing and incubating for 5 min at room temperature, the tubes were placed on a magnetic rack. Once the supernatant clarified, it was discarded, and tubes were washed twice with 400 μL of 70% freshly prepared ethanol each with an incubation time of 1 min after adding the ethanol. The pellet was then air‐dried (3–5 min), resuspended with 26 μL PCR‐grade water and incubated at room temperature for 5 min. After placing the tubes on a magnetic rack again for 2 min, 25 μL of clear supernatant was transferred into a new tube.

The DNA concentration of all four pools was assessed using the Qubit 4 Fluorometer (Thermo Fisher Scientific, Life Technologies, Carlsbad, CA, USA) with a dsDNA High‐Sensitivity Assay Kit (Thermo Fisher Scientific, Life Technologies, Carlsbad, CA, USA). To determine the amplicon length in the pools, they were loaded onto a High Sensitivity DNA Chip for measurement with the BioAnalyzer 2100 (Agilent Technologies, Santa Clara, USA). Amplicon length was expected to be around 490 bp; however, the peak can range due to variability of the ITS2 region. The library pool was supplemented with 5% PhiX Sequencing Control v3 (Illumina Inc., San Diego, CA, USA) for sequence diversity and loaded into a 500 cycle v2 reagent MiSeq cartridge (Illumina Inc., San Diego, CA, USA) as well as the sequencing primers read 1 and read 2, following Sickel et al. (2016). Sequencing was executed on a MiSeq system provided by the LMU Biocenter Genomics Service Unit. All 94 stigma samples were successfully amplified with an approximate amplicon target length of 490 bp, as well as the 12 positive controls. The 12 negative controls showed no amplicons on the gel.

### 2.4 Bioinformatic Processing

To prepare the sequencing data for subsequent analysis, VSEARCH v2.14.2 was employed, following the pipeline provided at https://github.com/chiras/metabarcoding_pipeline (Leonhardt et al. 2022). Paired ends of forward and reverse reads were joined, and all reads shorter than 150 bp were discarded. Furthermore, quality filtering (EE < 1) as described by Edgar and Flyvbjerg (2015), was applied, along with a de‐novo chimaera filtering using UCHIME3 (Edgar 2016). VSEARCH was also used to define Amplicon Sequence Variants (ASVs) (Edgar 2016). By using VSEARCH against an ITS2 reference database for plant species of the sampled region, reads were directly mapped with global alignments with an identity cut‐off threshold of 97%. The reference database, compiled with the BCdatabaser (Keller et al. 2020), was based on a list of German plant species and curated (Leonhardt et al. 2022; Quaresma et al. 2024). To classify still remaining reads without taxonomic allocation at this point, SINTAX (Edgar 2016) was used with a curated global reference database (Quaresma et al. 2024).

### 2.5 Revision of Sequences

Sample sequencing data were manually quality assessed by NMDs (Figure S8), sequencing depth and suspicious taxa composition. The most abundant ASV sequences (> 2000 reads) of these samples were checked manually with the Nucleotide Basic Local Alignment Tool (nBLAST; Altschul et al. 1990) and aligned with several reference sequences from BLAST and the reference database using Geneious Prime version 11.0.14.1+1 to reveal taxonomy classification errors during bioinformatic processing. Sequencing reads from the host species were also used to validate the field identification of the three *Ranunculus* species. In addition, the most abundant species in the samples apart from host species *R. acris* , *R. bulbosus* and *R. repens* , as well as species that had not been spotted on the plots during vegetation assessments, were also reviewed manually to check whether available information on these species' general distribution and blooming period argued for correct taxonomic classification. Distribution maps on FloraWeb provided by the Federal Agency for Nature Conservation in Germany and GBIF were consulted (https://www.gbif.org [13 January 2022]). If it was highly probable that a certain species had been misclassified, the corresponding sequence was manually checked and renamed or removed from the dataset if necessary. After manually checking the most abundant species concerning distribution area and blooming period, several classification changes were made, which are documented at: https://github.com/chiras/database‐curation/tree/main/corrections or in the R code removing further unclassified ASVs.

### 2.6 Data Analysis and Visualisation

All further analysis of sample data obtained by DNA metabarcoding were performed using R software, version 4.2.2 (R Core Team 2022) in RStudio (Posit team 2023) and managed with the phyloseq R package (McMurdie and Holmes 2013). Irrelevant taxa (fungi, algae) were filtered and multiple ASVs from the same species were collated. Additionally, low abundant species were filtered with a threshold of 0.08, based on the positive controls. Further packages used for data visualisation included ggplot2 (Wickham 2016), ggrepel (Kamil Slowikowski 2022), dplyr (Wickham et al. 2022), vegan (Oksanen et al. 2007; v2.6‐2) as well as tidyverse (Wickham et al. 2019), viridis (Garnier et al. 2021) and hrbrthemes (Bob Rudis 2020). Boxplots were created to depict the relative read abundances of the plant species found in the samples and controls (Figures S7 and S9). Bubble chart heatmaps were plotted to visualise the heterospecific pollen detected on *Ranunculus* stigma host species. To test whether plant species richness differed between low, intermediate and high land‐use intensity categories, we used a Kruskal–Wallis test with the function *‘Kruskal.test()’* (R Core Team 2022). Final datasets and R code can be found here: https://doi.org/10.5281/zenodo.12820055 (Werle et al. 2024a), https://doi.org/10.5281/zenodo.12819884 (Werle et al. 2024b).

### 2.7 Statistical Analysis

#### 2.7.1 Ranunculus Stigma Analysis

For further statistical analysis of *Plant Species Richness*, we used a subset of 74 samples, as plant data from three plots did not qualify for subsequent analysis. To test for collinearity of variables, we composed a correlation matrix using the functions ‘cor’ and ‘corrplot’ from the package (Corrplot; Wei and Simko 2021). Based on the correlation matrix (Figure S16), we included only *Plant species richness* in our models testing for effects on the richness of pollen on *Ranunculus* stigmas (*Richness stigma*). We checked for normal distribution using the Shapiro–Wilk normality test and Quantile–Quantile Plots. To test whether the respective *Ranunculus* host species ‘*Host*’ should be included as a fixed factor in the final models, we fitted three linear models with different fixed effects, either including or not including *Host* and its interaction with *Plant species richness* (Table S8). Models were compared using a likelihood ratio test and the Akaike information criterion (AIC), and interaction was included as a fixed factor in the final model.

We subsequently assessed the goodness of fit of the model using the packages ‘*effects*’ (Fox and Weisberg 2018a, 2018b) to visualise and simulate the effect of different distributions in explaining variation in residuals (Figure S17). By visualising the residuals of different models, we could show that model M3 (Table S8) had the best fit by adding a quadratic effect to *Plant species richness* (Figure S17B). We finally tested for a significant effect of random factors considering the nested design of the study, with host nested in plot nested in exploratory, using lm and lmer from the ‘*lme4’* package (Bates et al. 2015) as suggested by Zuur et al. (2009) (Table S9). Including these random effects did not significantly improve the model's explanatory values, which is why we did not include them in the final model. We further tested the effect of LUI using a stepwise regression based on AIC, with the stepwise selection method (step() function in R). The initial model included *Richness stigma* as the response variable, which was modelled as a function of the quadratic term of *Plant species richness*, the categorical variable *LUI*, their interaction, as well as the main effects of *Host* and *Plant Species Richness*, along with their interaction. The stepwise selection removed non‐significant terms, prioritising models with lower AIC values. Consequently, the final model used to analyse the effect of *Plant species richness* on *Richness stigma* was a linear model with a quadratic term for *Plant species richness* and an interaction of *Host* and *Plant species richness* included as a fixed factor.

## 3 Results

### 3.1 Heterospecific Pollen Detection

Overall, 94 stigma samples and 23 control samples were successfully sequenced, with a total of 3,597,026 quality filtered reads and an average throughput of 38,266 reads per sample (± 11,354) for the stigma samples.

**FIGURE 1:**
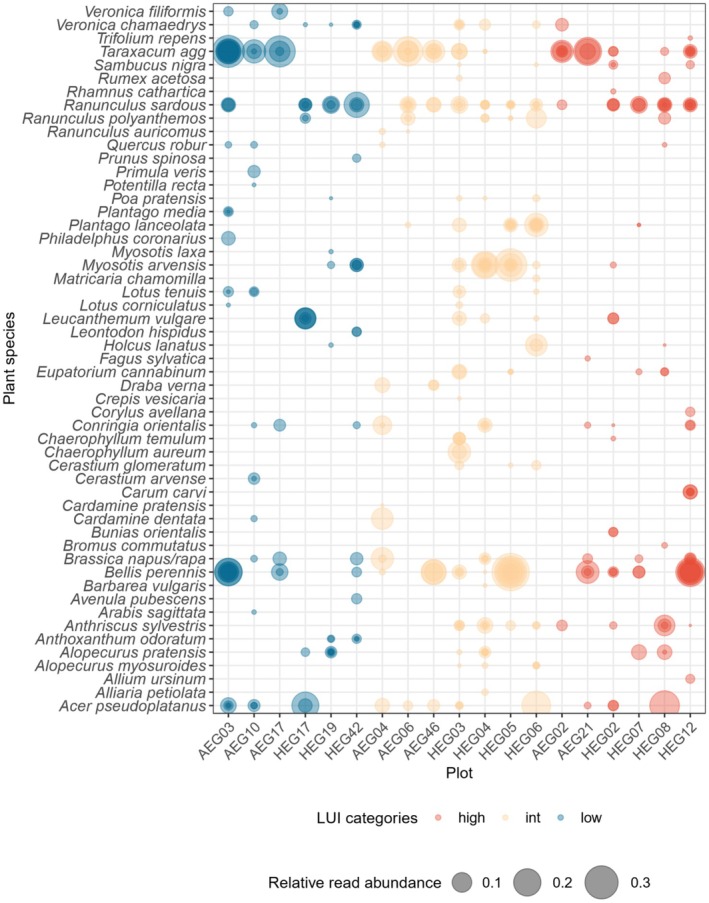
Bubble chart visualising the relative read abundance of plant species detected on 94 *Ranunculus* stigma samples from 19 plots via ITS2 DNA metabarcoding, collected in the Swabian Alb and Hainich‐Dün. For better resolution of heterospecific pollen, the reads for host plants ( *Ranunculus acris* , *R. bulbosus* and *R. repens* ) were removed. Samples from AEG07 were excluded as they were identified with a different host plant. Colours represent land‐use intensity index (LUI) categories, with blue for low LUI, yellow for intermediate (int) LUI and red for high LUI. Relative read abundance is visualised by the size of the bubble ranging from 0.1 to 0.4.

Including the host plant species, pollen from 56 different plant species was found on *Ranunculus* stigmas using ITS2 DNA metabarcoding (Figures 1 and S9, Table S4). In total, 40 plant species were detected in *R. acris* samples, 33 species in *R. bulbosus* samples, and 31 species in *R. repens* samples (Table S4). Pollen of the host species, *R. acris* , *R. bulbosus* or *R. repens*, was always the most abundant detected across samples, with a mean relative read abundance > 0.35 (Table S4). Apart from the host species, pollen of *Ranunculus sardous* (in 52 out of 94 samples), *Taraxacum agg*. (in 45 out of 94 samples) and *Bellis perennis* (in 37 out of 94 samples) was found in higher prevalences (Figure 1, Table S4). *R. sardous* , *T. agg*., as well as *B. perennis* pollen, was found across the complete range of land‐use intensity (Figure 1, Table S4). They did not only have the highest counts of detection but also the highest mean relative read abundance across samples (Figure 1, Table S4). Detection of *T. agg*. was highest on plots in the Swabian Alb during the first sampling period and lowest on plots in Hainich‐Dün during the second sampling period. Besides these, pollen of *Myosotis arvensis* and *Acer pseudoplatanus* was found in relatively high prevalence (20 out of 94 samples) particularly on intermediate and high LUI plots (Figure 1). In total, seven grass species were detected, with *Alopecurus pratensis* being the most abundant with 15 occurrences and a mean relative read abundance of 0.021 (Table S4). For all *Ranunculus* species, trees ( *Acer pseudoplatanus* , *Corylus avellana* , *Fagus sylvatica* and *Quercus robur* ) and shrubs ( *Philadelphus coronarius* , *Prunus spinosa* , *Rhamnus cathartica* and *Sambucus nigra* ), each with four species, were detected. Overall, pollen from a slightly higher number of different plants was found on *Ranunculus* stigmas on intermediate plots, with 32 unique plant species, followed by high LUI plots with 27 unique detections and low LUI plots with 26 unique detections (Figure 1, Table S1), although not statistically significant (Kruskal–Wallis: χ^2^ = 1.0553, df = 2, *p* value = 0.59).

### 3.2 Effects of Local Plant Species Richness on Heterospecific Pollen Composition and Richness on Ranunculus Stigmas

**FIGURE 2:**
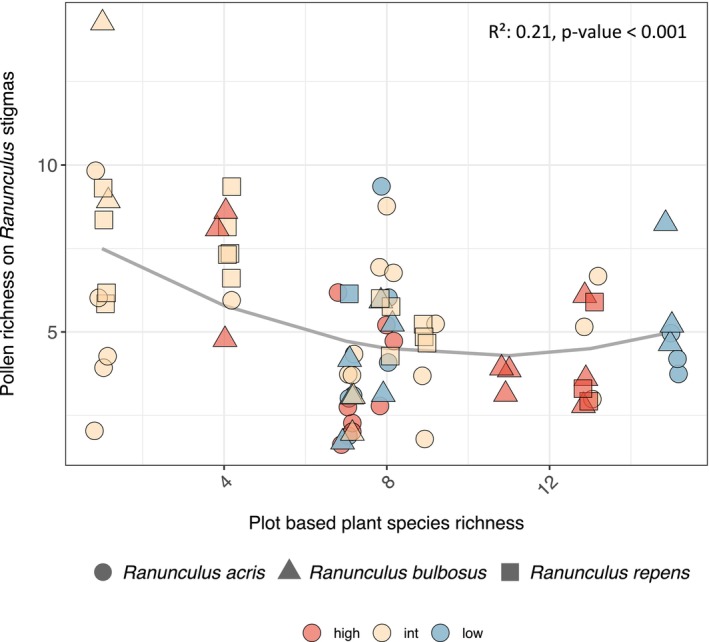
Relationship of pollen richness found on 76 *Ranunculus* stigma samples by using ITS2 DNA metabarcoding (*Richness stigma*) and plant species richness on surveyed plots. Different host species are visualised with different symbols and land‐use intensity index (LUI) categories are represented with blue for low LUI, yellow for intermediate (int) LUI and red for high LUI. A quadratic fit was added using a linear model: lm (Richness stigma ~ Plant species richness + *I* (Plant species richness^2^). Data points are jittered slightly for a better visualisation of overlaps. Multiple *R* ^2^ value and *p* value are presented in the upper right corner of the figure.

Overall, the richness of heterospecific pollen (*Richness stigma*) on stigmas of *Ranunculus* plants was significantly correlated with *Plant species richness*, both for the quadratic and the linear effect (Table 1, Figure 2), showing a nonlinear relationship. The interaction of *Host* and *Plant species richness* and *Host* itself did not show a significant effect (Table 1). We found that heterospecific pollen richness on *Ranunculus* stigma was higher on plots with low plant species richness, which corresponded to intermediate and some high LUI plots. This was followed by a slight decrease in heterospecific pollen richness as surrounding plant species richness increased across plots with varying levels of LUI. At the highest plant species richness levels, heterospecific pollen richness on *Ranunculus* stigma increased again (Figure 2).

**TABLE 1:** Type III analysis of variance table with Satterthwaite's method for the effect of surrounding p*lant species richness* on the richness of heterospecific pollen on the stigmas of three *Ranunculus* species (*Richness stigma*) collected on grassland plots differing in land‐use intensity index (LUI) in two different areas in Germany, for a subset of 76 samples.

Richness stigma	Df	Sum of Sq	Mean Sq	*F*‐value	Pr (> *F*)	Sign.
Plant species richness^2^	1	23.949	23.949	5.819	0.019	**
Host	2	24.481	12.241	2.974	0.058	ns.
Plant species richness	1	51.546	51.546	12.524	0.001	***
Host: Plant species richness	2	22.144	11.072	2.690	0.075	ns.

*Note:* *Plant species richness* entered as a quadratic effect and an interaction of h*ost* and linear *plant species richness* in the model, showing: Degrees of freedom (Df), sum of squares (Sum of Sq), mean squares (Mean Sq), *F*‐value and *p* value (Pr > *F*) with significance (Sign.) codes: ***0.001; **0.01; *0.05; ns > 0.05, and adjusted *R* ^2^ (Adj. *R* ^2^). Adj. *R* ^2^ = 0.24.

## 4 Discussion

### 4.1 Using DNA Metabarcoding to Infer Heterospecific Pollen Deposition Patterns

Our analysis of heterospecific pollen on *Ranunculus* stigmas demonstrates that DNA metabarcoding is a robust and effective method for identifying pollen deposited on stigmas, extending its previously established application to pollen collected by pollinators (Bell et al. 2023). This innovative approach offers, therefore, a more plant‐focused perspective on pollen transfer, pollinator behaviour (flower fidelity) and potentially the outcomes of pollination. Additionally, it allows for a more comprehensive understanding of the ecological interactions between different plant species and their pollinators.

However, a limitation of the method is that it primarily provides relative abundance data rather than absolute counts of pollen grains from each species. Although trends in pollen transfer can be inferred from relative read abundances of different species, the inability to directly quantify pollen grain counts limits insights into the volume of transferred pollen, which could be ecologically significant. In contrast, light microscopy offers a more quantitative measure of pollen grains; however, usually at lower taxonomic resolution (Milla et al. 2021). Therefore, the choice of methodology should align with the specific research question, balancing the need for taxonomic precision, quantitative data and ecological context.

### 4.2 Ecological Significance of Heterospecific Pollen Transfer

A high heterospecific pollen deposition might negatively impact plant reproductive success, for example, due to interference of conspecific pollen germination or reducing viable seed set (Morales and Traveset 2008). Even the same genotype might bring disadvantages to plant reproductive success when geitonogamy (transfer of pollen within the same plant individual (Johnson and Nilsson 1999)) occurs because of self‐incompatibility which could reduce seed set (Ashman and Arceo‐Gómez 2013). Geitonogamy might be more common in environments with reduced plant species richness, as, for example, seen on some intermediate LUI plots with a high richness of heterospecific pollen on stigmas.

Even when the heterospecific pollen itself does not result in successful pollination, for example, due to geitonogamy, the presence of heterospecific pollen on the stigma can still provide valuable information about pollinator behaviour in the surrounding landscape. Most pollinators are known to be either oligolectic or polylectic, and thus their behaviour can significantly affect the reproductive success of plants (Mitchell et al. 2009). Specifically, generalist pollinators are more likely to transfer heterospecific pollen between plant species, while specialists likely show higher flower fidelity (Hanoteaux et al. 2013) and may thus transfer less heterospecific pollen. For example, specialised oligolectic bees often visit a small range of closely related plant species, reducing the likelihood of heterospecific pollen transfer (Praz et al. 2008). However, even specialists can switch to a more generalist foraging behaviour when floral resources are scarce (Arstingstall et al. 2021; Newbold et al. 2018), which could result in increased heterospecific pollen deposition. The standard foraging behaviour of most pollinators comprises short distance flights between plants and the visitation of several flowers in sequence (Mitchell et al. 2009), which may impact plant mating and thus gene dispersal (Mitchell et al. 2009). In fact, the spatial distribution of plant species and the surrounding plant community can influence pollinator behaviour, especially foraging decisions, and may thus determine heterospecific pollen transfer and potentially lead to a higher or lower plant reproductive success (Geslin et al. 2014).

Our results show that the composition of heterospecific pollen changes with the diversity of plant species in the surrounding environment. Contrary to the parsimonious expectation that heterospecific pollen deposition would increase with plant species richness due to stochastic effects, we found that heterospecific pollen richness first decreased as plant diversity increased, before slightly increasing again for plots with the highest plant diversities (i.e., 11–15 plant species per quadrant). This trend persisted even though plants on plant species‐rich plots were likely visited by a greater number of pollinator species, as shown for the same sampling locations (Weiner et al. 2014). This apparent contradiction, that is, lower heterospecific pollen transfer despite higher plant diversity, may reflect increased pollinator fidelity in species‐rich environments. At low plant diversity sites with suboptimal choices, individual pollinators may need to visit more diverse plant species to meet their nutritional requirements, leading to greater heterospecific pollen transfer. Also, higher pollinator density and richness per flower (as seen for datasets from Weiner et al. (2014) and Parreño et al. (2025)) in such environments might increase competition and the frequency of heterospecific pollen transfer due to more frequent interspecies floral visits. Our findings thus support the hypothesis that higher plant species richness reduces heterospecific pollen transfer, likely due to stronger niche separation among pollinators, leading to greater modularity in pollination networks. This agrees with other studies showing that the diversity of plants within plant communities increased spatio‐temporal niche complementarity in plant–pollinator interactions (Venjakob et al. 2016). Indeed, higher plant species richness on a plot can provide more diverse floral resources across time and space, potentially allowing more pollinator species to specialise on different plant species, which subsequently reduces competition for the same floral resources (Blüthgen and Klein 2011; Hegland and Boeke 2006), and thereby reduces heterospecific pollen transfer.

Interestingly, we observed a slight increase in heterospecific pollen at plots with very high plant species richness. This finding contrasts with the overall trend of reduced heterospecific pollen transfer in species‐rich areas and may be explained by an increase in the probability of heterospecific pollen transfer beyond a certain threshold of plant and pollinator diversity due to the sheer abundance of pollinators, despite increased niche partitioning.

### 4.3 Temporal and Spatial Variability in Pollen Transfer

Variability in heterospecific pollen composition was observed even among plots within the same LUI category, with notable differences between study regions. This variability may result from differences in sampling periods or regional variations in plant community composition. For example, *Taraxacum agg*. was primarily found in plots from the Swabian Alb, sampled in early May, but was largely absent in later samples from Hainich‐Dün. Such differences in flowering phenology, rather than sampling effort, are likely to explain the observed variability in composition between regions.

Moreover, pollinator behaviour is likely additionally influenced by the spatial and temporal distribution, diversity and availability of floral resources found beyond the investigated plots. For instance, at low‐diversity sites, pollinators may need to increase foraging ranges and visit multiple plant species outside the measured areas to meet their nutritional requirements (Geslin et al. 2014; Hanoteaux et al. 2013; Devaux et al. 2014; Danner et al. 2016). This extended foraging behaviour could lead to higher levels of pollen exchange between distantly located plant species, especially in fragmented landscapes where floral resources are patchily distributed, which could increase the deposition of heterospecific pollen not just from the local immediate environment but also from further distances, diversifying the pollen transfer. On the contrary, in more plant species‐rich environments, pollinators might show a more selective behaviour, which might reduce the distance they travel between plants and thus limit heterospecific pollen transfer. Influencing factors on floral fidelity between plots of differing LUI could be floral diversity as well as floral density. Besides the plant community on plots, the spatial distribution, diversity and availability of plants in the surrounding environment may impact pollinator fidelity (Ebeling et al. 2008), as pollinators may have to collect pollen from multiple plant species to complete their nutritional needs and thus fly to floral resources outside of plots. This could also lead to an increased heterospecific pollen deposition at low diverse sites. However, we observed variability in heterospecific pollen composition even among plots with the same LUI. This may be explained by differences in sampling periods. The plots in the Swabian Alb were sampled at the beginning of May and the plots in Hainich‐Dün at the end of May. For example, Taraxacum agg. was found mostly in plots from the Swabian Alb, whereas Taraxacum agg. was largely absent in later sampling in plots from Hainich‐Dün. We assume that these temporal differences in flowering phenology account for the observed variability, rather than sampling effort.

### 4.4 Implications for Plant Reproductive Success

Local network structure of changing modularity coupled with the mobility of pollinators and their ability to adjust their foraging behaviour based on local resource availability may jointly explain the observed variability in heterospecific pollen deposition. We cannot distinguish between both factors, but, from a plant perspective, the proximate fact remains that increasing local plant diversity reduced heterospecific pollen deposition. Low heterospecific pollen transfer in our species‐rich communities likely benefits the plants, as they receive comparatively more conspecific pollen with a potentially positive effect for plant reproductive success. This reduction in heterospecific pollen may lead to improved pollen‐stigma compatibility, ensuring that stigmas are less likely to be clogged by pollen from unrelated species, which can inhibit fertilisation. Additionally, the reduced deposition of heterospecific pollen in plant species‐rich areas may enhance the efficiency of conspecific pollen transfer, which can improve the quality of fertilisation events and, over time, contribute to maintaining or enhancing genetic diversity within plant populations.

## 5 Conclusion

Our findings highlight the influence of surrounding plant species diversity on heterospecific pollen deposition. Lower stigma‐deposited heterospecific pollen diversity in plant species‐rich locations is likely a result of increasing local network modularity and smaller foraging ranges. Using DNA metabarcoding to detect heterospecific pollen offers a promising approach to investigating plant–pollinator interactions from a plant‐focused perspective. This method can provide novel insights into the effects of environmental factors on pollination dynamics, ultimately advancing our understanding of variation in plant reproductive success across different habitats and landscapes.

## Author Contributions

**Susanne Werle:** conceptualization (lead), data curation (lead), formal analysis (lead), investigation (equal), methodology (equal), project administration (equal), supervision (equal), validation (equal), visualization (lead), writing – original draft (lead), writing – review and editing (lead). **Anna Preußner:** conceptualization (supporting), data curation (supporting), formal analysis (equal), investigation (equal), methodology (equal), validation (supporting), visualization (supporting), writing – original draft (supporting), writing – review and editing (supporting). **Kenneth Kuba:** data curation (supporting), methodology (supporting), supervision (supporting), validation (supporting), writing – original draft (supporting). **Sara Diana Leonhardt:** conceptualization (equal), formal analysis (supporting), funding acquisition (lead), methodology (supporting), project administration (supporting), supervision (equal), validation (supporting), writing – original draft (supporting), writing – review and editing (supporting). **Alexander Keller:** conceptualization (supporting), data curation (supporting), formal analysis (supporting), funding acquisition (lead), project administration (supporting), resources (supporting), supervision (supporting), visualization (supporting), writing – original draft (supporting), writing – review and editing (supporting).

## Conflicts of Interest

There are no conflicts of interest.

## Supporting information


Data S1.

## Data Availability

All raw ITS2 DNA metabarcoding data generated by this study are deposited in the SRA (BioProject PRJNA1137278) and available on NCBI via the following link: https://www.ncbi.nlm.nih.gov/bioproject/PRJNA1137278. Vegetation assessments are publicly available in the Biodiversity Exploratories Information System (BExIS) (http://doi.org/10.17616/R32P9Q) with the dataset id: 31556 (Parreno et al. 2021) and available for download here: https://doi.org/10.5281/zenodo.13145226 (Werle, Kuba, et al. 2024). ITS2 DNA metabarcoding data table of Ranunculus stigma, including metadata, is available on BExIS with the dataset id: 31837 (Werle 2024) and can be downloaded with all R codes on zenodo: https://doi.org/10.5281/zenodo.12820055 (Werle et al. 2024a) and https://doi.org/10.5281/zenodo.12819884 (Werle et al. 2024b). All data are permanently archived in BExIS.
