# Supplementary material for: Unveiling Heterospecific Pollen Deposition in Ranunculus Plants Along a Land‐Use Gradient Through DNA Metabarcoding
Source: Ecol Evol. 2025 Mar 27;15(4):e71184. doi: 10.1002/ece3.71184 (PMC11949568; doi:10.1002/ece3.71184)
Supplement: Supplementary file 1 — Data S1. [file ECE3-15-e71184-s001.docx]

**Supplemental materials**

**Unveiling heterospecific pollen deposition in *Ranunculus* plants along a land-use gradient through DNA metabarcoding**

Susanne Werle*^1,2^, Anna Preußner*^2^, Kenneth Kuba^1^, Sara Diana Leonhardt**^1^, and Alexander Keller**^2^

^1^ Plant-Insect Interactions, TUM School of Life Science Systems, Technical University of Munich (TUM), Freising, Germany. E-mail: susanne.werle@tum.de. ORCID: 0000-0001-8524-276X

^2^ Cellular and Organismic Networks, Faculty of Biology, LMU Munich, Munich, Germany

* Shared first authorship

** Shared last authorship

Corresponding author: Susanne Werle, susanne.werle@tum.de

**Supplementary material – part 1: *Ranunculus* stigma sampling methods**

*Sampling sites and sample collection*

Both sampling locations within the Biodiversity Exploratories are shown in Figure S1. Figure S2 and S3 display the distribution of sampling sites within the Swabian Alb (ALB) and Hainich-Dün (HAI) exploratory. Coordinates for each sampled plot can be found in Table S1. Flower assessments were conducted on each plot by using 10 1x1 m quadrants, identifying and counting flowering plant species as well as open flowers (Figure S4). Number of samples differed per Exploratory (Figure S5) which was caused by cold and wet weather conditions and thus less flowers flowering in the beginning of May 2022.


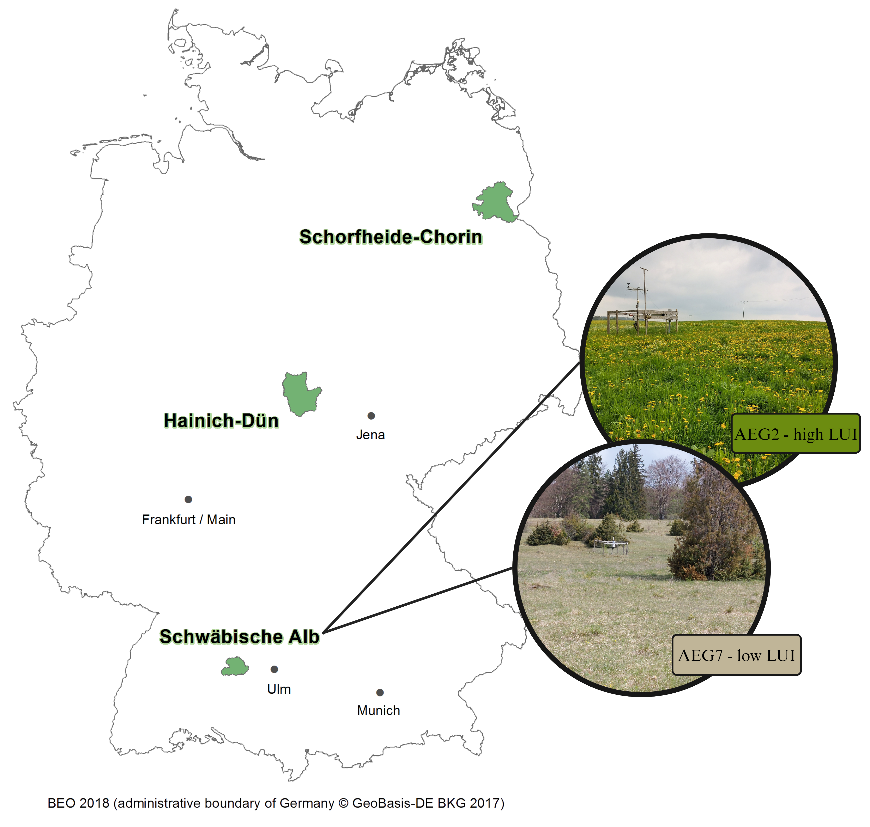


**Figure S1** Locations of both sampling regions, Swabian Alb (ALB) and Hainich-Dün (HAI) within the Biodiversity Exploratories. Two examples of images are shown with a high and low land-use intensity index (LUI) for two plots in the ALB. Created with BioRender.com.

**
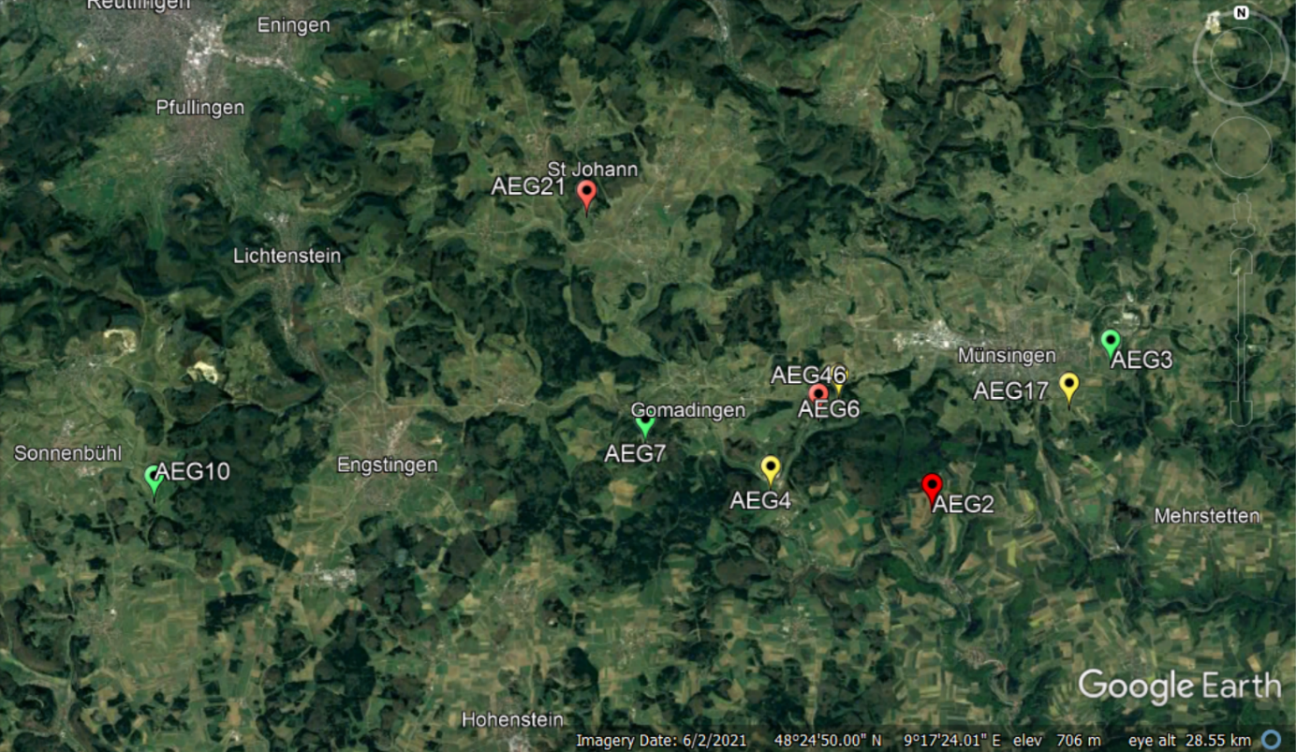
Figure S2** Plots (AEG 03, AEG 02, AEG 04, AEG 06, AEG 07, AEG 10, AEG 17, AEG 21, AEG 46) subject to different land-use intensities (LUI) sampled in the Swabian Alb (ALB) (Google Earth, 2022, plot markers by Andreas Ostrowski). Ranunculus stigmas were sampled on three low LUI (green), three intermediate LUI (yellow) and three high LUI (red) grassland plots in the ALB.

**
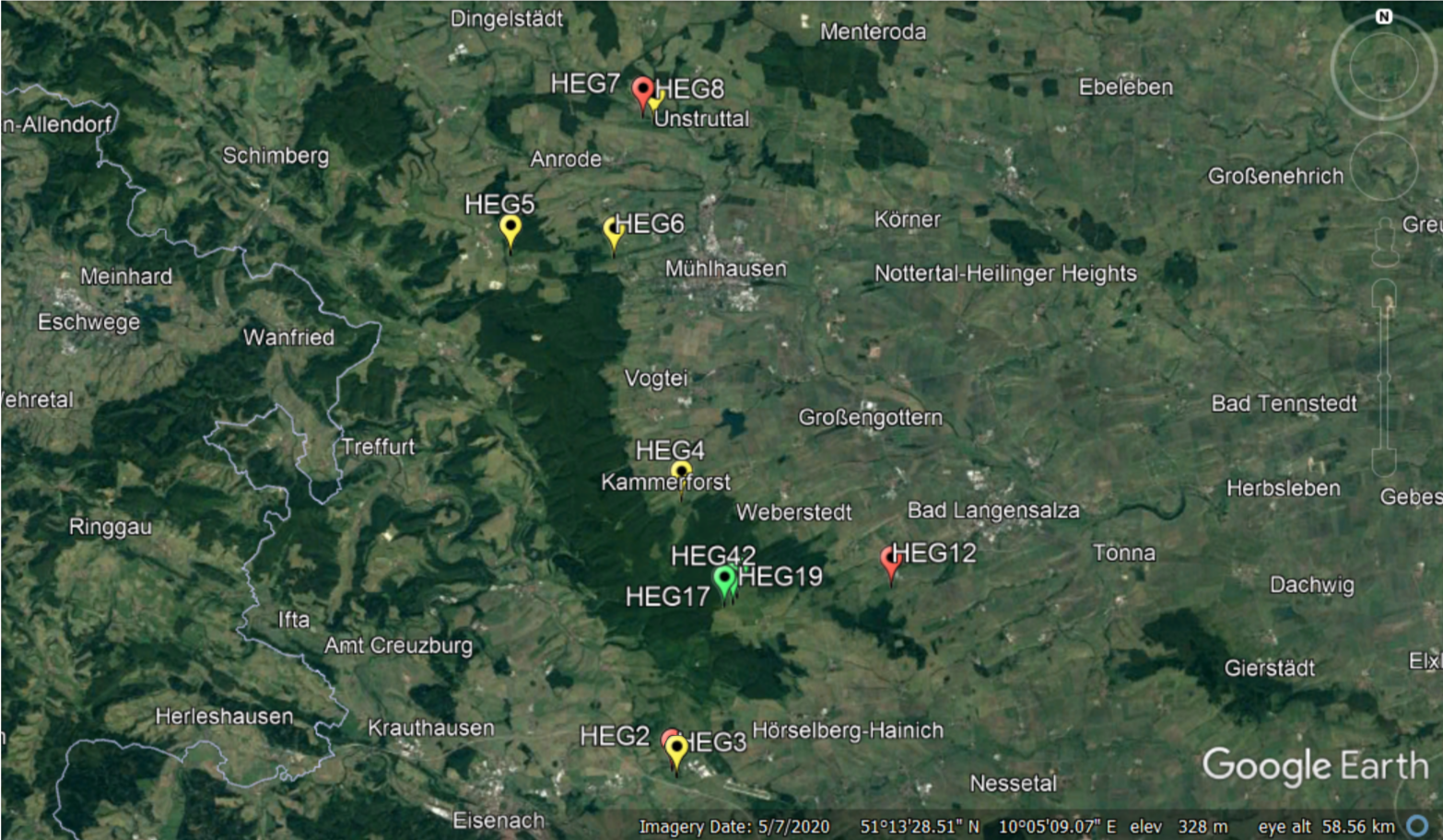
**

**Figure S3** Plots (HEG 02, HEG 03, HEG 04, HEG 05, HEG 06, HEG 07, HEG 08, HEG 12, HEG 17, HEG 19, HEG 42) subject to different land-use intensities (LUI) sampled in the Hainich-Dün (HAI) exploratory (Google Earth, 2022, plot markers by Andreas Ostrowski). *Ranunculus* stigmas were sampled on three low LUI (green), five intermediate LUI (yellow) and three high LUI (red) grassland plots in Hainich-Dün.

*
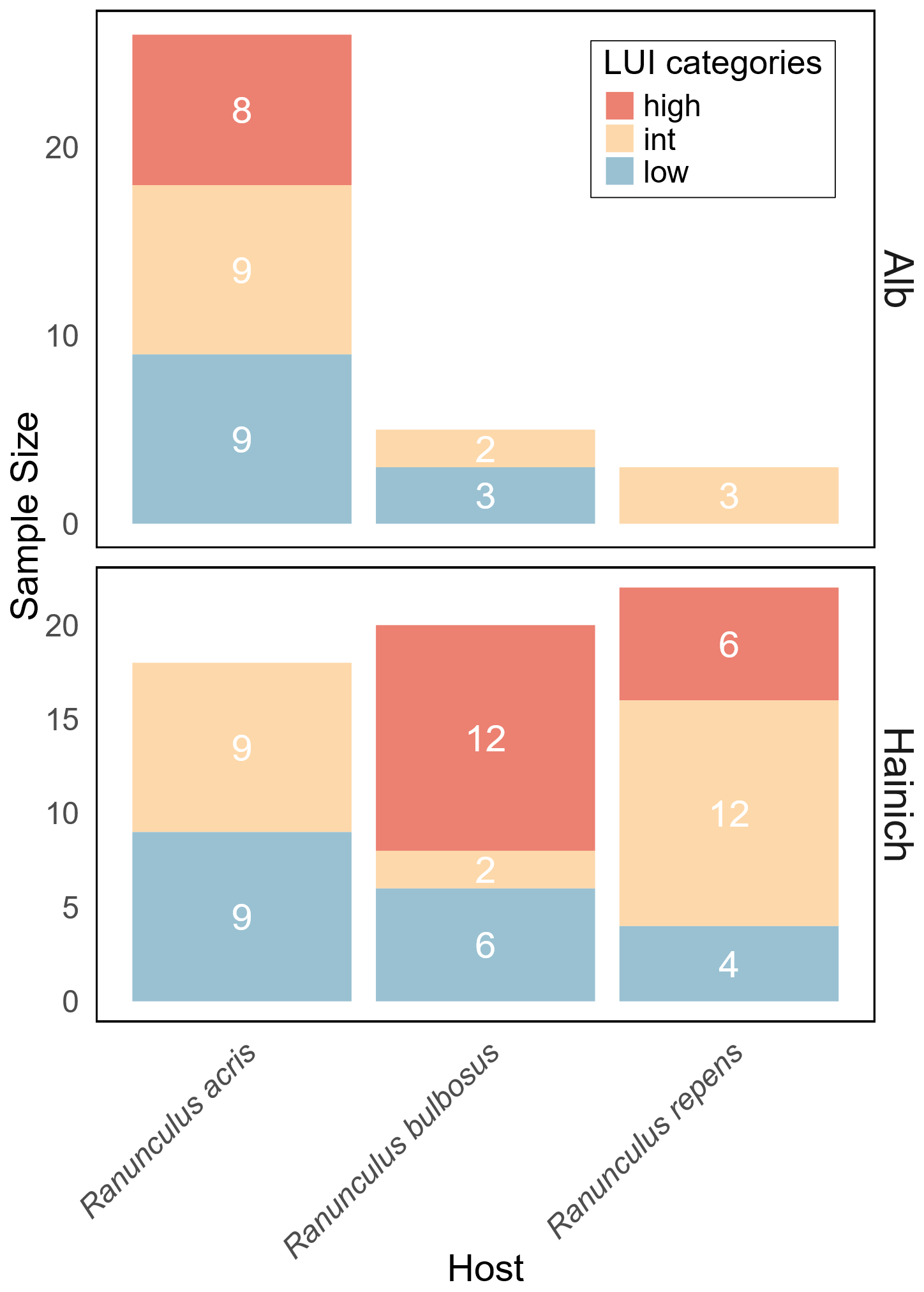

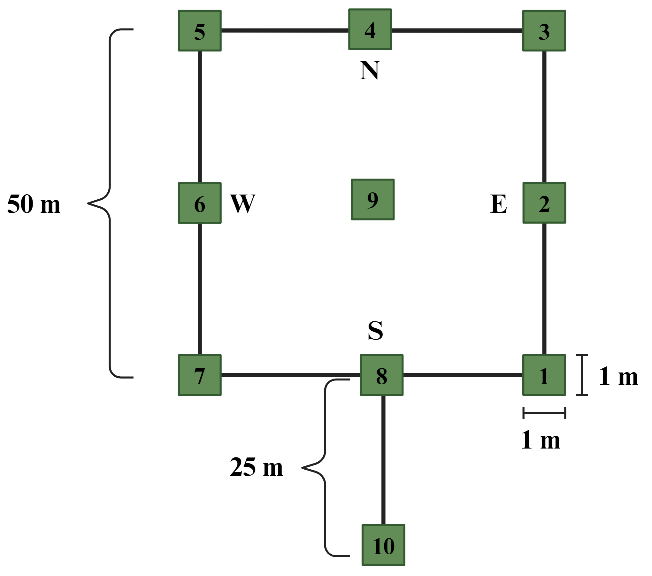
***Figure S4** Visual representation of ten 1x1 meter quadrants for vegetation assessments. We measured flowering plant species richness in each plot, counting the plant species and the number of open flowers in each quadrant. Created with BioRender.com.

**Figure S5** We collected a total of 94 stigma samples from *Ranunculus acris*, *R. bulbosus*, and *R. repens* on plots with varying land-use intensities (LUI) in the Swabian Alb (ALB) and Hainich-Dün (HAI) exploratory regions. Samples from each LUI category are color-coded: green for low LUI plots (ALB:12; HAI:19), blue for intermediate LUI plots (ALB:14; HAI:23), and red for high LUI plots (ALB:8; HAI:18). Each sample, representing a patch, contains a pool of 16 – 20 stigmas.

**Table S1** Coordinates and Land-use intensity index (LUI) category for all sampled plots, including the number of plant species detections (N plants) on *Ranunculus* stigmas summarised by plot. LUI categories were set with the following ranges based on percentile calculations: low category with LUI values between 0 and 1.44; intermediate category with values between 1.45 and 2.13 and a high category with values between 2.14 and 3.85. On low LUI plots 26 unique plant species were detected on *Ranunculus* stigmas, 32 on intermediate LUI plots and 27 on high LUI plots.

| Plot | Latitude | Longitude | LUI category | N plants |
| --- | --- | --- | --- | --- |
| AEG02 | 48.3768572731 | 9.4727841247 | high | 7 |
| AEG03 | 48.4088814832 | 9.5323787503 | low | 12 |
| AEG04 | 48.3808884549 | 9.4188891246 | int | 11 |
| AEG06 | 48.4012621530 | 9.4416781078 | int | 9 |
| AEG10 | 48.3787128703 | 9.2126008478 | low | 13 |
| AEG17 | 48.3993230386 | 9.5183688727 | low | 6 |
| AEG21 | 48.4422038842 | 9.3572193006 | high | 7 |
| AEG46 | 48.3969714231 | 9.4348888005 | int | 7 |
| HEG02 | 51.0007489248 | 10.4300100103 | high | 13 |
| HEG03 | 50.9980914934 | 10.4329488763 | int | 24 |
| HEG04 | 51.1133623520 | 10.4361803786 | int | 18 |
| HEG05 | 51.2158944134 | 10.3225267190 | int | 11 |
| HEG06 | 51.2149350553 | 10.3912208733 | int | 18 |
| HEG07 | 51.2735825557 | 10.4104125695 | high | 7 |
| HEG08 | 51.2712574671 | 10.4179445812 | high | 12 |
| HEG12 | 51.0772200104 | 10.5758197075 | high | 13 |
| HEG17 | 51.0705045490 | 10.4704560664 | low | 9 |
| HEG19 | 51.0732375079 | 10.4732197941 | low | 11 |
| HEG42 | 51.0694413682 | 10.4647839624 | low | 13 |

*Improvement for sample processing*

We conducted a preliminary experiment to optimize pollen detachment from stigmas. We therefore tested two approaches:

1) Single stigmas were placed in 2 mL microcentrifuge tubes filled with 1x Phosphate-buffered saline (PBS; VWR Chemicals, Ohio, USA) and vortexed for 1 min to avoid damage

2) Multiple stigmas were combined in one tube with 1x PBS, vortexed for 1 min, and examined under a stereo microscope.

Method 2 proved most effective; vortexing one patch of pooled stigmas together (n = 16 – 20) removed pollen effectively (Figure S6B). When washing single stigmas, some pollen remained. PBS drops from each tube were examined in Petri dishes to confirm pollen removal (see Figure S6C).


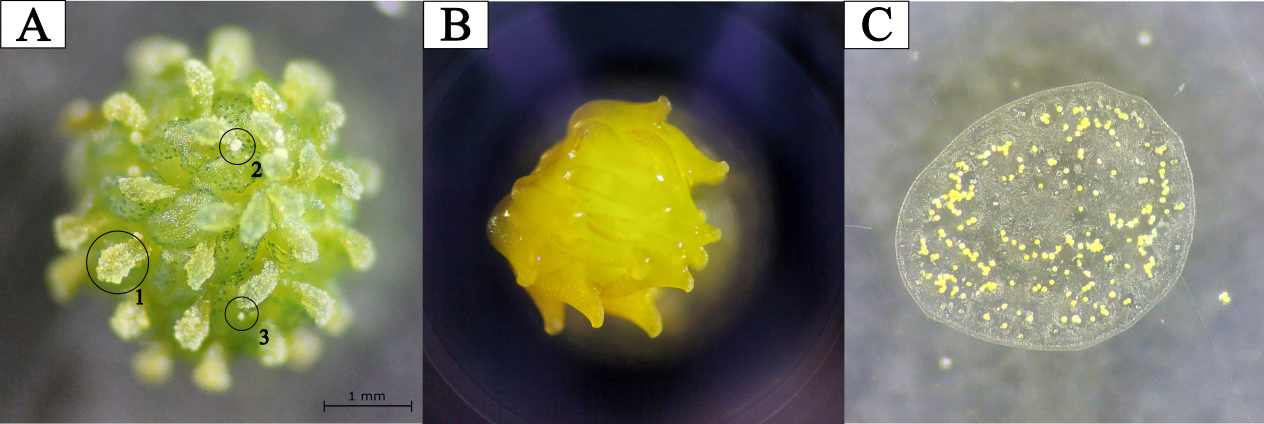


**Figure S6** Ranunculus acris stigma viewed under a stereo microscope. A) displays a stigma before washing, with (1) many small yellow pollen grains, as well as small (2) and medium sized (3) white pollen found on the stigma’s surface. B) shows a washed Ranunculus acris stigma and C) a dried drop of pollen dissolved in Phosphate-buffered saline after vortexing several stigmas together, with substantial amounts of pollen grains washed off.

*Sequencing methods and results*

Primers used for DNA amplification of samples and controls are listed in Table S2 and Index sequences for each sample in Table S3.

*Sequencing primers*

**Table S2** Primer sequences for dual-indexing for ITS2 DNA metabarcoding by Sickel et al. (2016). Sequences used for forward and reverse primers, with XXXXXXXX is a place holder for the variable index sequences, which are listed in Table S3.

| Primer | Primer sequence |
| --- | --- |
| F primer sequence | 5’- AATGATACGGCGACCACCGAGATCTACAC XXXXXXXX  CCTGGTGCTG GT ATGCGATACTTGGTGTGAAT-3’ |
| R primer sequence | 5’- CAAGCAGAAGACGGCATACGAGAT XXXXXXXX  AGTCAGTCAG CC TCCTCCGCTTATTGATATGC-3’ |

**Table S3** Primer index sequences. Unique forward and reverse index combination for each sample and control applied for the DNA metabarcoding protocol by Sickel *et al.* (2016).

| Sample name | Forward index | F. index sequence | Reverse index | R. index sequence |
| --- | --- | --- | --- | --- |
| A4_RA1 | SB503 | AGAGTCAC | SA710 | GTCTGCTA |
| A4_RA2 | SB503 | AGAGTCAC | SA701 | CGAGAGTT |
| A4_RA3 | SA505 | TCATCGAG | SB706 | GTAACGAG |
| A7_RP1 | SA505 | TCATCGAG | SB701 | CTCGACTT |
| A7_RP2 | SA505 | TCATCGAG | SB702 | CGAAGTAT |
| A7_RP3 | SA505 | TCATCGAG | SB703 | TAGCAGCT |
| A17_RA1 | SB503 | AGAGTCAC | SA709 | ACTACGAC |
| A17_RA2 | SA506 | CGTGAGTG | SB704 | TCTCTATG |
| A17_RA3 | SA506 | CGTGAGTG | SB705 | GATCTACG |
| A2_RA1_1 | SA506 | CGTGAGTG | SB701 | CTCGACTT |
| A2_RA2_1 | SA506 | CGTGAGTG | SB702 | CGAAGTAT |
| A3_RA1 | SA505 | TCATCGAG | SB707 | ACGTGCGC |
| A3_RA2 | SA505 | TCATCGAG | SB708 | ATAGTACC |
| A3_RA3 | SA505 | TCATCGAG | SB709 | GCGTATAC |
| A6_RA1 | SB503 | AGAGTCAC | SA702 | GACATAGT |
| A6_RA2 | SA506 | CGTGAGTG | SB707 | ACGTGCGC |
| A6_RA3 | SA506 | CGTGAGTG | SB708 | ATAGTACC |
| A3_RB1 | SA505 | TCATCGAG | SB710 | TGCTCGTA |
| A3_RB2 | SA505 | TCATCGAG | SB711 | AACGCTGA |
| A3_RB3 | SA505 | TCATCGAG | SB712 | CGTAGCGA |
| A21_RA1 | SA507 | GGATATCT | SB705 | GATCTACG |
| A21_RA2 | SB503 | AGAGTCAC | SA704 | ACTCACTG |
| A21_RA3 | SA507 | GGATATCT | SB708 | ATAGTACC |
| A46_RA1 | SA506 | CGTGAGTG | SB711 | AACGCTGA |
| A46_RA2 | SA506 | CGTGAGTG | SB712 | CGTAGCGA |
| A46_RA3 | SA507 | GGATATCT | SB701 | CTCGACTT |
| A46_RB1 | SA506 | CGTGAGTG | SB709 | GCGTATAC |
| A46_RB2 | SA506 | CGTGAGTG | SB710 | TGCTCGTA |
| A6_RR1 | SB503 | AGAGTCAC | SA708 | CGAGCGAC |
| A6_RR2 | SA507 | GGATATCT | SB703 | TAGCAGCT |
| A6_RR3 | SA507 | GGATATCT | SB704 | TCTCTATG |
| A10_RA1 | SA507 | GGATATCT | SB709 | GCGTATAC |
| A10_RA_Mix | SA507 | GGATATCT | SB711 | AACGCTGA |
| A10_RA2 | SA507 | GGATATCT | SB710 | TGCTCGTA |
| A10_RA3 | SA507 | GGATATCT | SB712 | CGTAGCGA |
| A2_RA1_2 | SA508 | GACACCGT | SB702 | CGAAGTAT |
| A2_RA2_2 | SA508 | GACACCGT | SB703 | TAGCAGCT |
| A2_RA3 | SA508 | GACACCGT | SB704 | TCTCTATG |
| H3_RR1 | SB503 | AGAGTCAC | SA705 | TGAGTACG |
| H3_RA2 | SA508 | GACACCGT | SB709 | GCGTATAC |
| H3_RA3 | SA508 | GACACCGT | SB710 | TGCTCGTA |
| H4_RR4 | SB504 | TACGAGAC | SB709 | GCGTATAC |
| H4_RR5 | SB504 | TACGAGAC | SB710 | TGCTCGTA |
| H2_RB1 | SA508 | GACACCGT | SB705 | GATCTACG |
| H2_RB2 | SA508 | GACACCGT | SB706 | GTAACGAG |
| H2_RB3 | SA508 | GACACCGT | SB707 | ACGTGCGC |
| H3_RB1 | SA508 | GACACCGT | SB711 | AACGCTGA |
| H3_RB2 | SA508 | GACACCGT | SB712 | CGTAGCGA |
| H4_RR1 | SB505 | ACGTCTCG | SA701 | CGAGAGTT |
| H4_RR2 | SB505 | ACGTCTCG | SA702 | GACATAGT |
| H4_RR3 | SB505 | ACGTCTCG | SA703 | ACGCTACT |
| H12_RB1 | SB507 | GATCGTGT | SA704 | ACTCACTG |
| H12_RB2 | SB507 | GATCGTGT | SA705 | TGAGTACG |
| H7_RB1 | SB506 | TCGACGAG | SA707 | TAGTCTCC |
| H7_RB2 | SB506 | TCGACGAG | SA708 | CGAGCGAC |
| H7_RB3 | SB506 | TCGACGAG | SA709 | ACTACGAC |
| H8_RB1 | SB507 | GATCGTGT | SA701 | CGAGAGTT |
| H8_RB2 | SB504 | TACGAGAC | SB707 | ACGTGCGC |
| H8_RB3 | SB507 | GATCGTGT | SA703 | ACGCTACT |
| H12_RR1 | SB504 | TACGAGAC | SB712 | CGTAGCGA |
| H12_RR2 | SB507 | GATCGTGT | SA708 | CGAGCGAC |
| H12_RR3 | SB507 | GATCGTGT | SA709 | ACTACGAC |
| H5_RR1 | SB505 | ACGTCTCG | SA707 | TAGTCTCC |
| H5_RR2 | SB505 | ACGTCTCG | SA708 | CGAGCGAC |
| H5_RR3 | SB505 | ACGTCTCG | SA709 | ACTACGAC |
| H5_RA1 | SB505 | ACGTCTCG | SA710 | GTCTGCTA |
| H5_RA2 | SB505 | ACGTCTCG | SA711 | GTCTATGA |
| H5_RA3 | SB505 | ACGTCTCG | SA712 | TATAGCGA |
| H8_RR1 | SB506 | TCGACGAG | SA710 | GTCTGCTA |
| H8_RR2 | SB506 | TCGACGAG | SA711 | GTCTATGA |
| H8_RR3 | SB506 | TCGACGAG | SA712 | TATAGCGA |
| H17_RA1 | SB507 | GATCGTGT | SA710 | GTCTGCTA |
| H17_RA2 | SB507 | GATCGTGT | SA711 | GTCTATGA |
| H17_RA3 | SB507 | GATCGTGT | SA712 | TATAGCGA |
| H19_RA1 | SB508 | GTCAGATA | SA705 | TGAGTACG |
| H19_RA3 | SB508 | GTCAGATA | SA707 | TAGTCTCC |
| H42_RA1 | SB508 | GTCAGATA | SA711 | GTCTATGA |
| H42_RA2 | SB508 | GTCAGATA | SA712 | TATAGCGA |
| H42_RA3 | SB505 | ACGTCTCG | SB702 | CGAAGTAT |
| H6_RA1 | SB506 | TCGACGAG | SA704 | ACTCACTG |
| H6_RA2 | SB506 | TCGACGAG | SA705 | TGAGTACG |
| H6_RA3 | SB504 | TACGAGAC | SB711 | AACGCTGA |
| H17_RB1 | SB504 | TACGAGAC | SB706 | GTAACGAG |
| H17_RB2 | SB508 | GTCAGATA | SA702 | GACATAGT |
| H17_RB3 | SB508 | GTCAGATA | SA703 | ACGCTACT |
| H42_RB1 | SB501 | CTACTATA | SB702 | CGAAGTAT |
| H42_RB2 | SB501 | CTACTATA | SB703 | TAGCAGCT |
| H42_RB3 | SB501 | CTACTATA | SB704 | TCTCTATG |
| H17_RR1 | SB508 | GTCAGATA | SA704 | ACTCACTG |
| H19_RR1 | SB505 | ACGTCTCG | SB701 | CTCGACTT |
| H19_RR2 | SB508 | GTCAGATA | SA709 | ACTACGAC |
| H19_RR3 | SB508 | GTCAGATA | SA710 | GTCTGCTA |
| H6_RR1 | SB506 | TCGACGAG | SA701 | CGAGAGTT |
| H6_RR2 | SB506 | TCGACGAG | SA702 | GACATAGT |
| H6_RR3 | SB504 | TACGAGAC | SB708 | ATAGTACC |
| H19_RA2 | SB508 | GTCAGATA | SA706 | CTACGCAG |
| H12_RB3 | SB507 | GATCGTGT | SA706 | CTACGCAG |
| H4_RA3 | SB505 | ACGTCTCG | SA706 | CTACGCAG |
| E01WE_H12 | SA508 | GACACCGT | SA712 | TATAGCGA |
| E02WP_E5 | SA505 | TCATCGAG | SB705 | GATCTACG |
| E02WP_F6 | SA506 | CGTGAGTG | SB706 | GTAACGAG |
| E02WP_G7 | SA507 | GGATATCT | SB707 | ACGTGCGC |
| E02WP_H8 | SA508 | GACACCGT | SB708 | ATAGTACC |
| E03WE_C3 | SB503 | AGAGTCAC | SA703 | ACGCTACT |
| E03WE_E5 | SB505 | ACGTCTCG | SA705 | TGAGTACG |
| E03WE_F6 | SB506 | TCGACGAG | SA706 | CTACGCAG |
| E03WE_G7 | SB507 | GATCGTGT | SA707 | TAGTCTCC |
| E03WE_H8 | SB508 | GTCAGATA | SA708 | CGAGCGAC |
| E04WE_A1 | SB501 | CTACTATA | SB701 | CTCGACTT |
| E04WE_B2 | SB502 | CGTTACTA | SB702 | CGAAGTAT |
| E02P_E4 | SA505 | TCATCGAG | SB704 | TCTCTATG |
| E02P_F3 | SA506 | CGTGAGTG | SB703 | TAGCAGCT |
| E02P_G2 | SA507 | GGATATCT | SB702 | CGAAGTAT |
| E02P_H1 | SA508 | GACACCGT | SB701 | CTCGACTT |
| E03P_C6 | SB503 | AGAGTCAC | SA706 | CTACGCAG |
| E03P_D5 | SB504 | TACGAGAC | SA705 | TGAGTACG |
| E03P_E4 | SB505 | ACGTCTCG | SA704 | ACTCACTG |
| E03P_F3 | SB506 | TCGACGAG | SA703 | ACGCTACT |
| E03P_G2 | SB507 | GATCGTGT | SA702 | GACATAGT |
| E03P_H1 | SB508 | GTCAGATA | SA701 | CGAGAGTT |
| E04P_A8 | SB501 | CTACTATA | SB708 | ATAGTACC |

*Revision of sequences*

*Lathyrus latifolius* sequence MG234585 on BLAST was mislabelled and could be identified as *Taraxacum agg*., therefore the taxonomic information was changed in the data. Furthermore, sequences classified as *Brassica rapa* and *Brassica napus* during bioinformatic processing could not be determined reliably on species level, so they were collated *to Brassica rapa/napus*. To conclude the revision*, Ranunculus damascenus* and *Ranunculus gouanii* sequences were combined to *Ranunculus spc*.

*Quality control - Negative and positive controls during extraction and PCR*

Control samples were included for specific steps for the steps in the wet lab, like DNA extraction and polymerase chain reaction (PCR). For the positive controls, *Holcus lanatus* DNA extraction was performed identically to the stigma samples. Positive controls serve as the most important quality control during PCR, as they will show cross-contamination of samples more likely. Negative controls were prepared in two types: 'neg_ext' (for negative extraction controls) and 'neg_pcr' (for negative PCR controls). For 'neg_ext' controls, DNA extraction was performed using the same food kit as for the stigma samples, with PCR-grade water replacing the biological sample. The 'neg_pcr' controls were added into the PCR scheme and processed along the stigma samples to detect cross contamination. They consisted of PCR-grade water and did not undergo DNA extraction. Negative controls are mostly important for the lab process and exclude any contamination within reagents during extraction and PCR.

*Negative extraction and PCR controls*

Analysis of all control samples, as displayed in Figure S7, showed that, besides *Ranunculus* itself, the most abundant plant species within the negative extraction controls was *Holcus lanatus*, which is the species added to the positive controls. *Acer pseudoplatanus* and *Plantago lanceolata* could be detected in positive and negative controls as well as samples from the Swabian Alb and Hainich. In total, 26 plant species were found throughout the negative controls. For ten negative control samples, we found higher amounts of the host species *R. acris* (Mean rel. abund. 0.280, SD = 0.182*), R. bulbosus* (Mean rel. abund. 0.283, SD = 0.147*) and R. repens* (Mean rel. abund. 0.278, SD = 0.070*)*. Further species found within the negative controls, but not in any other stigma samples were *Ajuga reptans*, *Alopecurus pratensis*, *Arabidopsis arenosa*, *Papaver rhoeas*, *Robinia pseudoacacia*, *Salix alba,* and *Trifolium pratense*.

*Positive PCR controls*

*Crepis vesicaria*, *Securigera varia* and *Rhinanthus alectorolophus* were only found in positive controls, however, not in any stigma samples. Only for one positive control sample three other ASVs were detected. Two of those had an abundance below 1 %. One ASV for *Plantago lanceolata* had 2.8 % relative read abundance and was also detected in the other positive controls. This is most probably a contamination within the positive control itself.


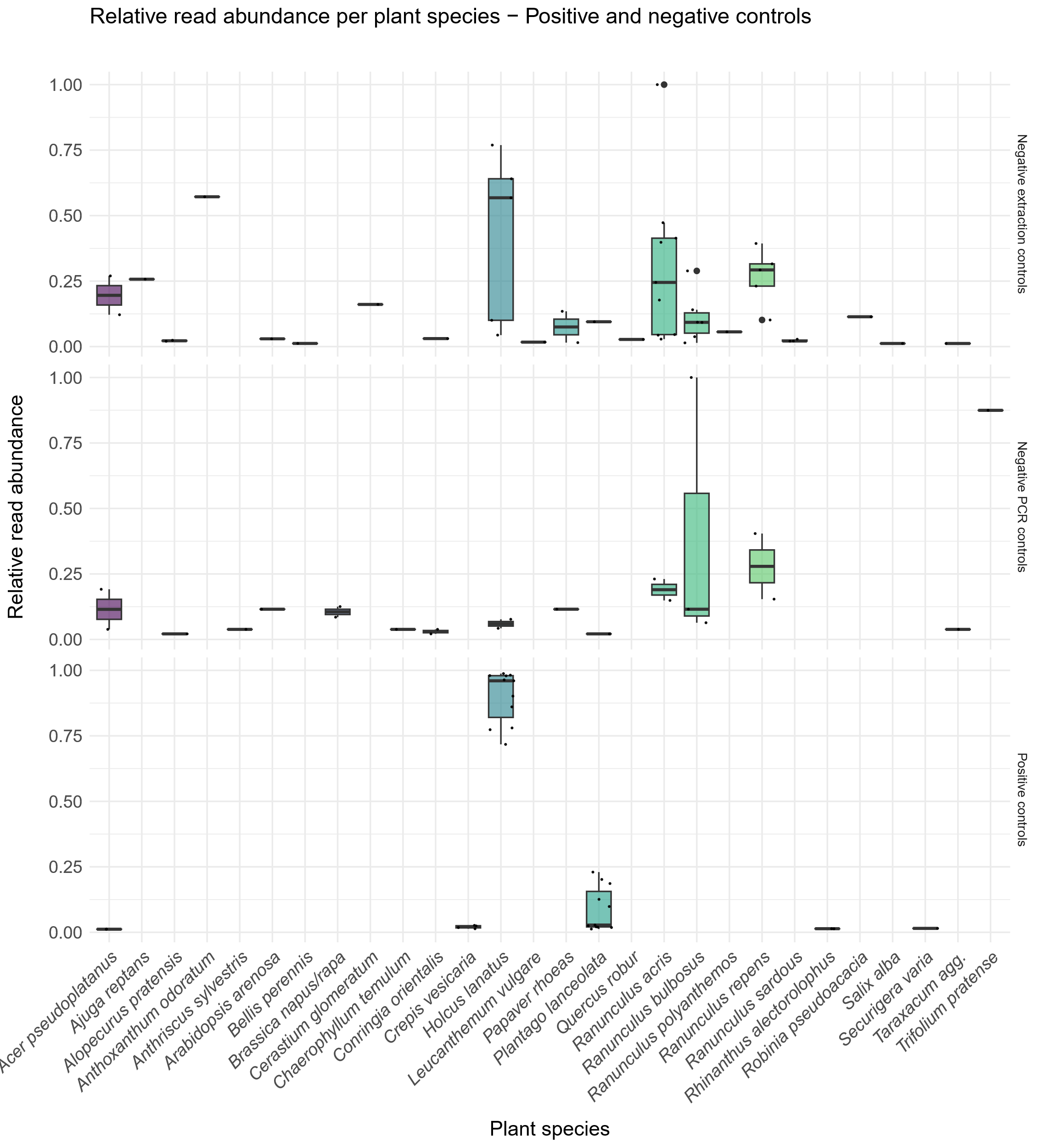


**Figure S7** Relative read abundances for amplified plant species, which were detected in: 1) negative extraction controls, 2) negative polymerase chain reaction (PCR) controls and 3) in positive controls. Negative extraction controls serve as a control during DNA extraction and are processed along with a sample without adding any sample tissue. PCR negative controls are included during PCR preparation and handled just the same as the samples with the difference of adding DNA free water instead of extracted DNA. Positive controls serve as the most important quality control during PCR, and all contain the DNA of Holcus lanatus.

Relative read abundance of plant species amplified in extraction- and PCR controls.

To validate, that samples clustered according to their host species, we used a Non-metric MultiDimentional Scaling (NMDS) for visualisation purposes (Figure S8). We used the function *ordinate* within the package phyloseq (McMurdie & Holmes 2013) in R to obtain the NMDS ordination, using the following settings: “ method="NMDS", "bray",k=2, trymax=500” and plotted it by using ggplot2 (Wickham 2016). The results showed a clear separation between the host species, always being the most abundant one, with mostly 15,000 – 40,000 reads per ASV.


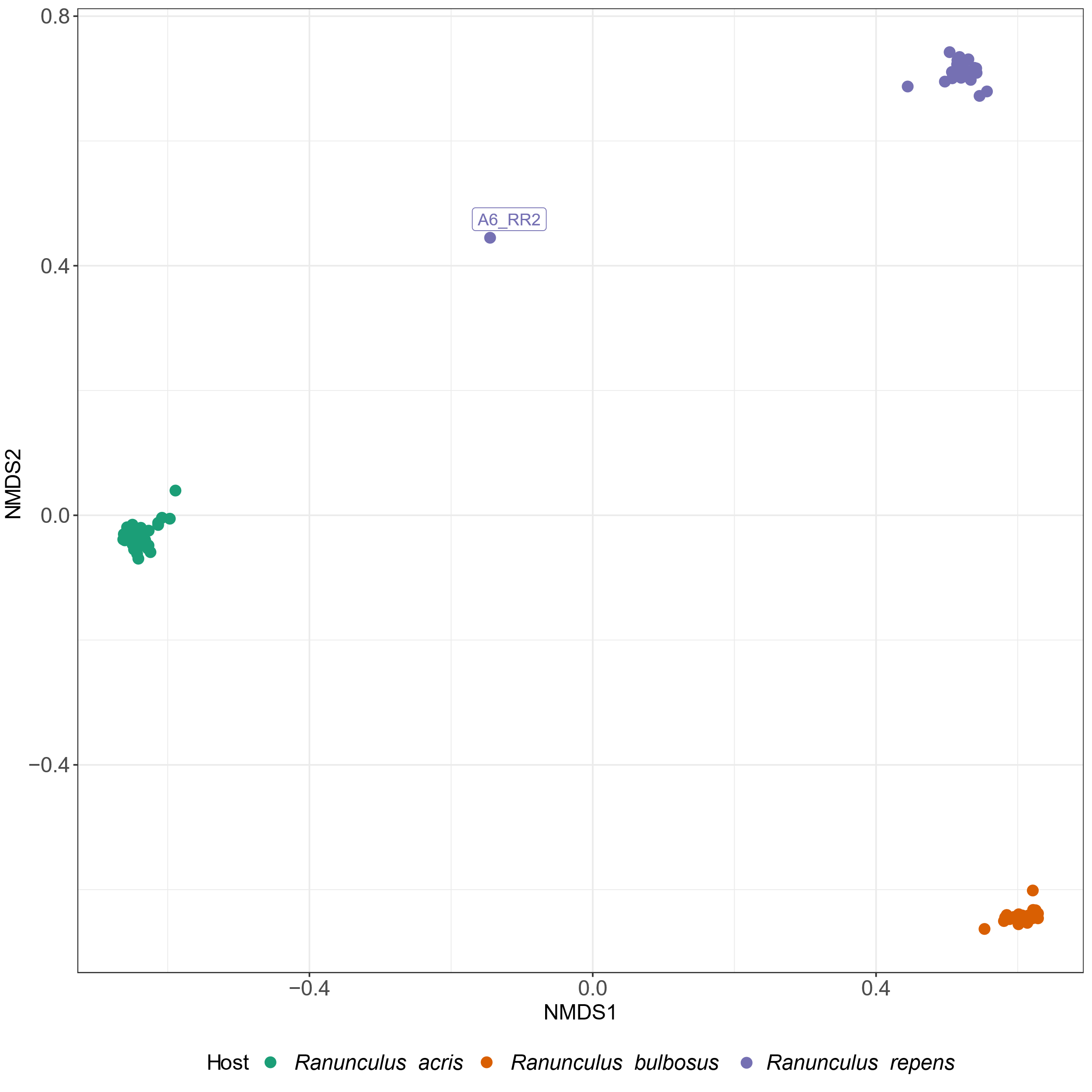


**Figure S8** Non-metric MultiDimentional Scaling (NMDS), to visualise host species clustering together. Shown are all successfully sequenced stigma samples. All stigma samples cluster according to their host species, except for sample A6_RR2, which has a mix of Ranunculus acris and Ranunculus repens sequences.

*Heterospecific pollen detection on Ranunculus stigmas*

Mean relative read abundances and detections for all three host species of stigma samples, can be found in Table S4.

*
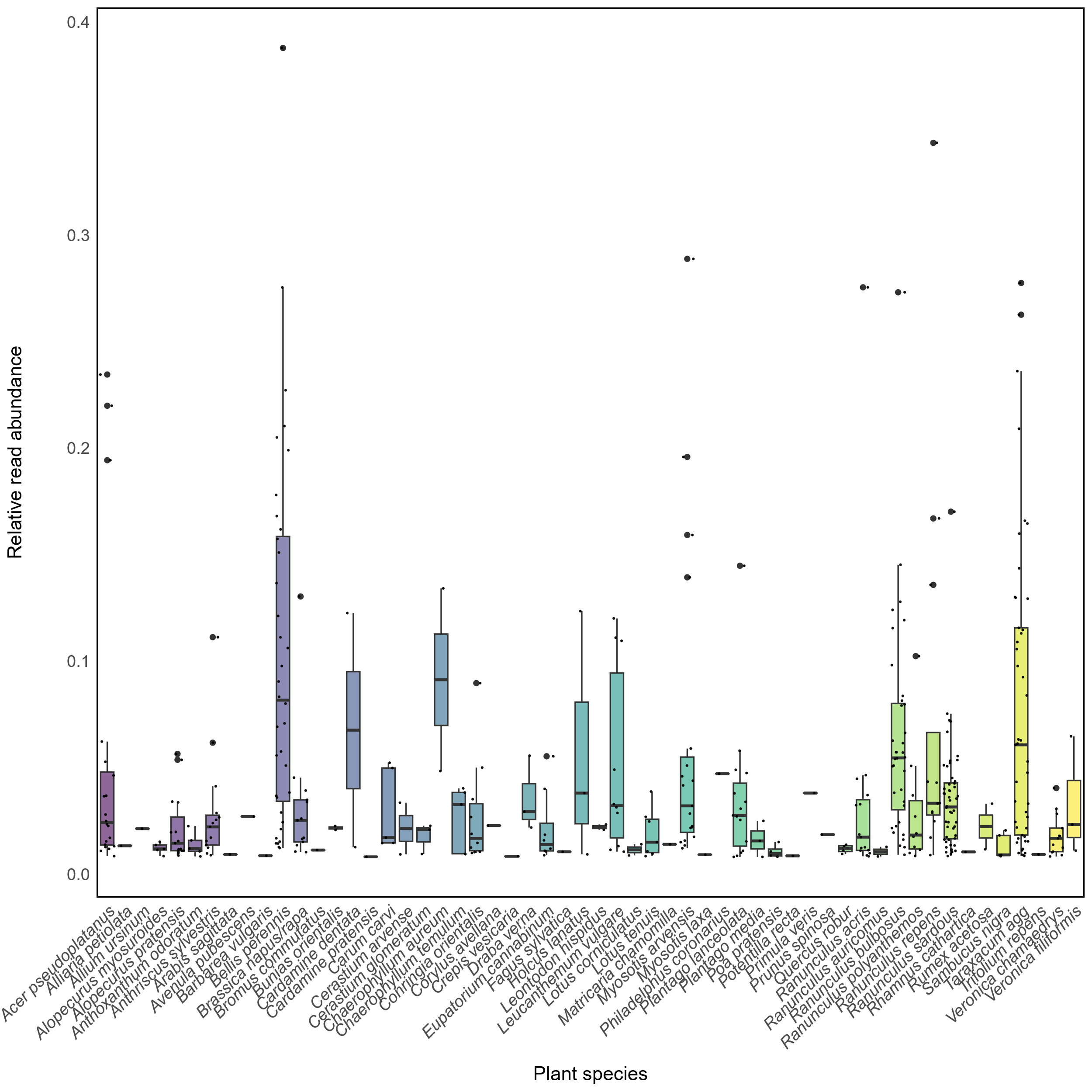
*

**Figure S9** Relative read abundances of all detected plant species found on all *Ranunculus* stigma samples. For better visualisation relative read abundances for *Ranunculus acris*, *R. bulbosus* and *R. repens* were removed with a threshold of 0.44, as those represented the highest number of reads for the host species.

**Table S4** Mean relative read abundances and standard deviation (SD) for overall plant species detections (n = 56) for all stigma samples using ITS2 DNA metabarcoding. Number of detections for each host species are given, as well as the sum of all unique species detections per host at the end of the table. The number of detections over all samples for a specific plant species is given in column ‘count’. *Ranunculus* host species have the highest relative read abundances, as well as the highest counts, as their own DNA from the stigmas was very prominent in all samples.

| Plant species | Mean relative read abundance | SD relative read abundance | Detections *R. acris* | Detections *R. bulbosus* | Detections *R. repens* | Count |
| --- | --- | --- | --- | --- | --- | --- |
| *Ranunculus acris* | 0.586 | 0.044 | 44 | 3 | 13 | 60 |
| *Ranunculus repens* | 0.466 | 0.052 | 8 | 4 | 25 | 37 |
| *Ranunculus bulbosus* | 0.35 | 0.046 | 11 | 25 | 25 | 61 |
| *Bellis perennis* | 0.101 | 0.014 | 18 | 13 | 6 | 37 |
| *Chaerophyllum aureum* | 0.091 | 0.043 | 1 | 1 | 0 | 2 |
| *Taraxacum agg* | 0.08 | 0.011 | 27 | 12 | 6 | 45 |
| *Cardamine dentata* | 0.068 | 0.055 | 2 | 0 | 0 | 2 |
| *Myosotis arvensis* | 0.063 | 0.017 | 10 | 3 | 7 | 20 |
| *Holcus lanatus* | 0.057 | 0.034 | 2 | 0 | 1 | 3 |
| *Acer pseudoplatanus* | 0.054 | 0.016 | 10 | 8 | 2 | 20 |
| *Leucanthemum vulgare* | 0.049 | 0.013 | 5 | 4 | 2 | 11 |
| *Philadelphus coronarius* | 0.047 | NA | 0 | 1 | 0 | 1 |
| *Primula veris* | 0.038 | NA | 1 | 0 | 0 | 1 |
| *Draba verna* | 0.036 | 0.009 | 3 | 0 | 0 | 3 |
| *Plantago lanceolata* | 0.036 | 0.01 | 7 | 3 | 5 | 15 |
| *Ranunculus sardous* | 0.035 | 0.004 | 5 | 25 | 22 | 52 |
| *Veronica filiformis* | 0.033 | 0.016 | 3 | 0 | 0 | 3 |
| *Brassica napus/rapa* | 0.031 | 0.008 | 7 | 1 | 6 | 14 |
| *Carum carvi* | 0.03 | 0.009 | 0 | 3 | 2 | 5 |
| *Ranunculus polyanthemos* | 0.03 | 0.009 | 0 | 1 | 9 | 10 |
| *Anthriscus sylvestris* | 0.028 | 0.007 | 6 | 4 | 6 | 16 |
| *Avenula pubescens* | 0.027 | 0.007 | 1 | 0 | 0 | 1 |
| *Conringia orientalis* | 0.027 | NA | 7 | 0 | 4 | 11 |
| *Chaerophyllum temulum* | 0.026 | 0.007 | 2 | 3 | 0 | 5 |
| *Corylus avellana* | 0.023 | NA | 0 | 1 | 0 | 1 |
| *Bunias orientalis* | 0.022 | 0.011 | 0 | 2 | 0 | 2 |
| *Leontodon hispidus* | 0.022 | 0.001 | 2 | 0 | 0 | 2 |
| *Rumex acetosa* | 0.022 | 0.001 | 0 | 1 | 1 | 2 |
| *Allium ursinum* | 0.021 | 0.006 | 0 | 1 | 0 | 1 |
| *Alopecurus pratensis* | 0.021 | 0.004 | 4 | 2 | 9 | 15 |
| *Cerastium arvense* | 0.021 | 0.012 | 2 | 0 | 0 | 2 |
| *Eupatorium cannabinum* | 0.021 | NA | 2 | 4 | 2 | 8 |
| *Lotus tenuis* | 0.019 | 0.004 | 4 | 2 | 1 | 7 |
| *Prunus spinosa* | 0.019 | NA | 0 | 1 | 0 | 1 |
| *Cerastium glomeratum* | 0.018 | 0.003 | 1 | 1 | 1 | 3 |
| *Veronica chamaedrys* | 0.018 | 0.004 | 7 | 3 | 3 | 13 |
| *Plantago media* | 0.016 | 0.005 | 1 | 2 | 0 | 3 |
| *Anthoxanthum odoratum* | 0.014 | NA | 4 | 1 | 0 | 5 |
| *Matricaria chamomilla* | 0.014 | 0.002 | 0 | 0 | 1 | 1 |
| *Alliaria petiolata* | 0.013 | NA | 0 | 0 | 1 | 1 |
| *Sambucus nigra* | 0.013 | 0.003 | 0 | 3 | 2 | 5 |
| *Alopecurus myosuroides* | 0.012 | 0.001 | 2 | 0 | 3 | 5 |
| *Quercus robur* | 0.012 | 0.001 | 2 | 2 | 0 | 4 |
| *Bromus commutatus* | 0.011 | 0.003 | 0 | 0 | 1 | 1 |
| *Lotus corniculatus* | 0.011 | NA | 2 | 0 | 0 | 2 |
| *Poa pratensis* | 0.011 | 0.002 | 1 | 0 | 3 | 4 |
| *Ranunculus auricomus* | 0.011 | 0.002 | 2 | 0 | 0 | 2 |
| *Fagus sylvatica* | 0.01 | NA | 1 | 0 | 0 | 1 |
| *Rhamnus cathartica* | 0.01 | NA | 0 | 1 | 0 | 1 |
| *Arabis sagittata* | 0.009 | NA | 1 | 0 | 0 | 1 |
| *Barbarea vulgaris* | 0.009 | NA | 0 | 0 | 1 | 1 |
| *Myosotis laxa* | 0.009 | NA | 1 | 0 | 0 | 1 |
| *Potentilla recta* | 0.009 | NA | 1 | 0 | 0 | 1 |
| *Trifolium repens* | 0.009 | NA | 0 | 0 | 1 | 1 |
| *Cardamine pratensis* | 0.008 | NA | 1 | 0 | 0 | 1 |
| *Crepis vesicaria* | 0.008 | NA | 0 | 0 | 1 | 1 |
| **Sum of unique species detections per host species** |  |  | **40** | **33** | **31** |  |

**Supplementary material – part 2: Reanalysis of plant pollinator visitations data obtained from Christiane Weiner**

We assumed that more pollinators were visiting *Ranunculus* plant species on plots with a high plant and pollinator species diversity and typically found on plots with low LUI (Weiner *et al.* 2014) with subsequent effects on the deposition of heterospecific pollen on stigma.

To test this assumption, we used the published datasets from Christiane Weiner obtained from the online data platform Biodiversity Instrumentation Project (BExIS dataset ID 15086, 4981, and 4963 (Weiner *et al.* 2019a; Weiner *et al.* 2019b; Weiner *et al.* 2022), which has been collected in 2008. From dataset 15086 the Shannon index as well as species richness was calculated once for pollinators found only on *Ranunculus* plant species (*Richness/Shannon pollinators on Ranunculus*) and for pollinators found over the total number of flowering plant species (*Richness/Shannon pollinators total*), for the Swabian Alb and Hainich-Dün using the vegan package (Jari Oksanen *et al.* 2022). The two variables (*Richness/Shannon*) were highly correlated for all flowering plants as well as for the subset of only *Ranunculus* species (Figure S10), which is why we used *Richness pollinators* *on Ranunculus* and *Richness pollinators total* in subsequent analyses. In addition, the number of *Ranunculus* individuals found on the sampled plots were extracted. Additionally, from datasets 4981 and 4963, *Plant species richness* per plot was calculated. We fitted a linear mixed-effects model (LMM) to investigate the effect of *Plant species richness* interacting with *LUI* (explanatory variables) on pollinator diversity on *Ranunculus* plants (response variable) using the package “*lme4”* (Bates *et al.* 2015). LUI was entered as categorical variables, with values between 0 and 1.5 for low LUI plots, values between > 1.5 and <= 2.0 for intermediate LUI plots and values between > 2.0 and <= 3.5 for high LUI plots. We first tested whether Exploratory (1|EXPLO) or the total number of *Ranunculus* individuals (1|Total Ranunculus Ind.) needed to be added as a random factor using the function “ranef”. Random effects were only detected for *Total Ranunculus Ind.*, but not for EXPLO and thus Exploratory was not included in the final model.

Using data from Weiner *et al.* (2014), we plotted *Richness pollinators on* *Ranunculus* against *Plant species richness* and tested for a significant correlation using a linear model with land-use categories interacting with *Plant species richness*. On high and intermediate LUI plots, the number of pollinator species visiting *Ranunculus* flowers increased with high *Plant species richness*, whereas we found no correlation for low LUI plots (Figure S12). In addition, we plotted *Ranunculus* density against plot-based *Plant species richness* and found low *Ranunculus* density on plots with low *Plant species richness* and increasing *Ranunculus* density with increasing *Plant species richness* on plots (Figure S11). We found the number of *Ranunculus* individuals per plot to increase with increasing surrounding plant species diversity (Figure S11). Interestingly, species richness of pollinators on *Ranunculus* plants was affected by the interaction between LUI and *Plant species richness* on plots (Figure S12, Table S5). It increased with *Plant species richness* for plots with high and intermediate LUI, while there was no correlation with *Plant species richness* for low LUI plots (Figure S12 Table S5). In summary, the final model explains 12.1% of the variance with its fixed effects and 63.2% of the variance when both fixed and random effects are considered.


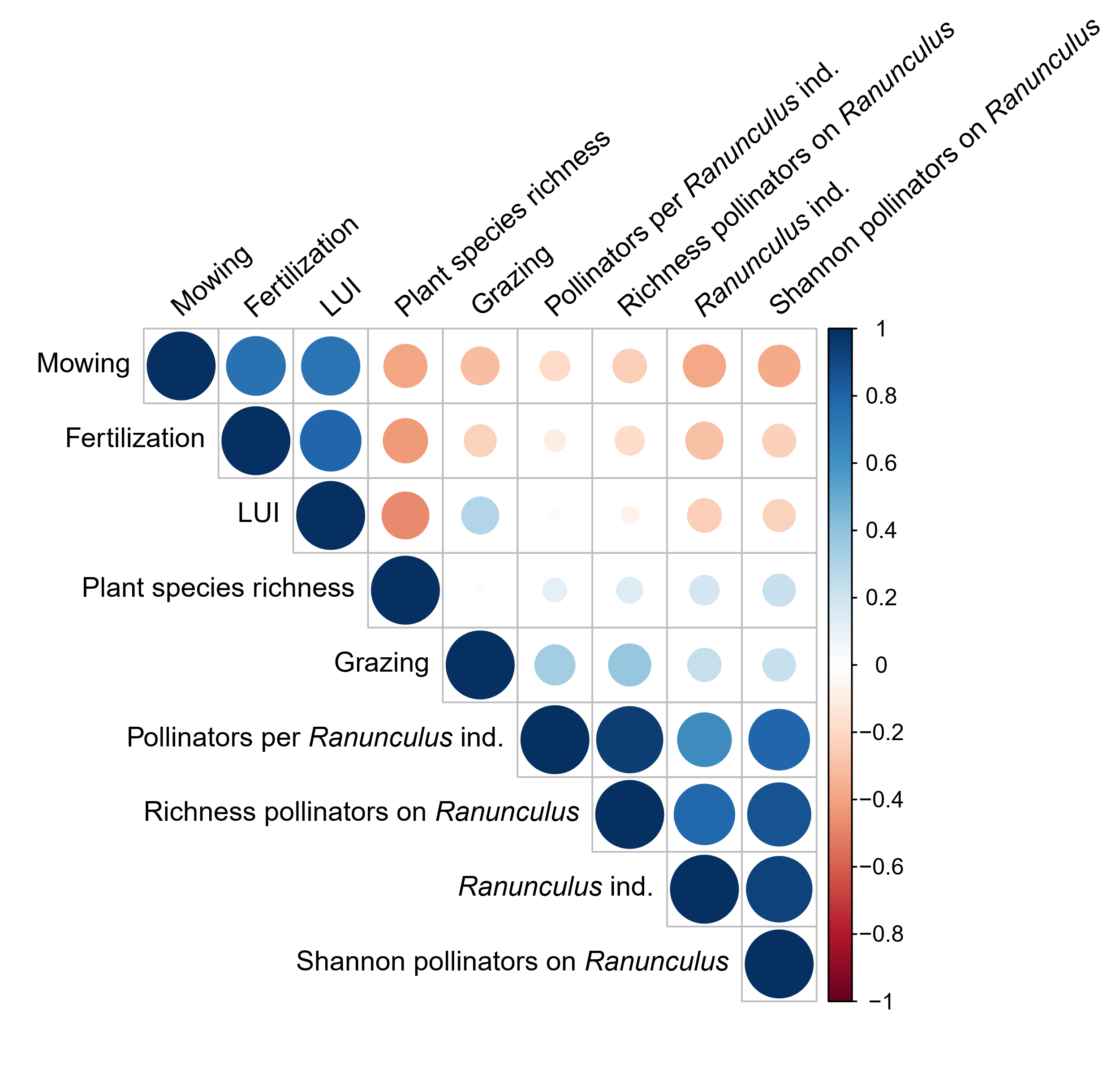


**Figure S10** Correlation matrix of response and explanatory variables for data from Christane Weiner 2008 (BExIS dataset ID 15086, 4981, and 4963 (Weiner et al. 2019a; Weiner et al. 2019b; Weiner et al. 2022)), with a heatmap-like visualisation of the correlation coefficients where the variables are ordered based on hierarchical clustering.

**
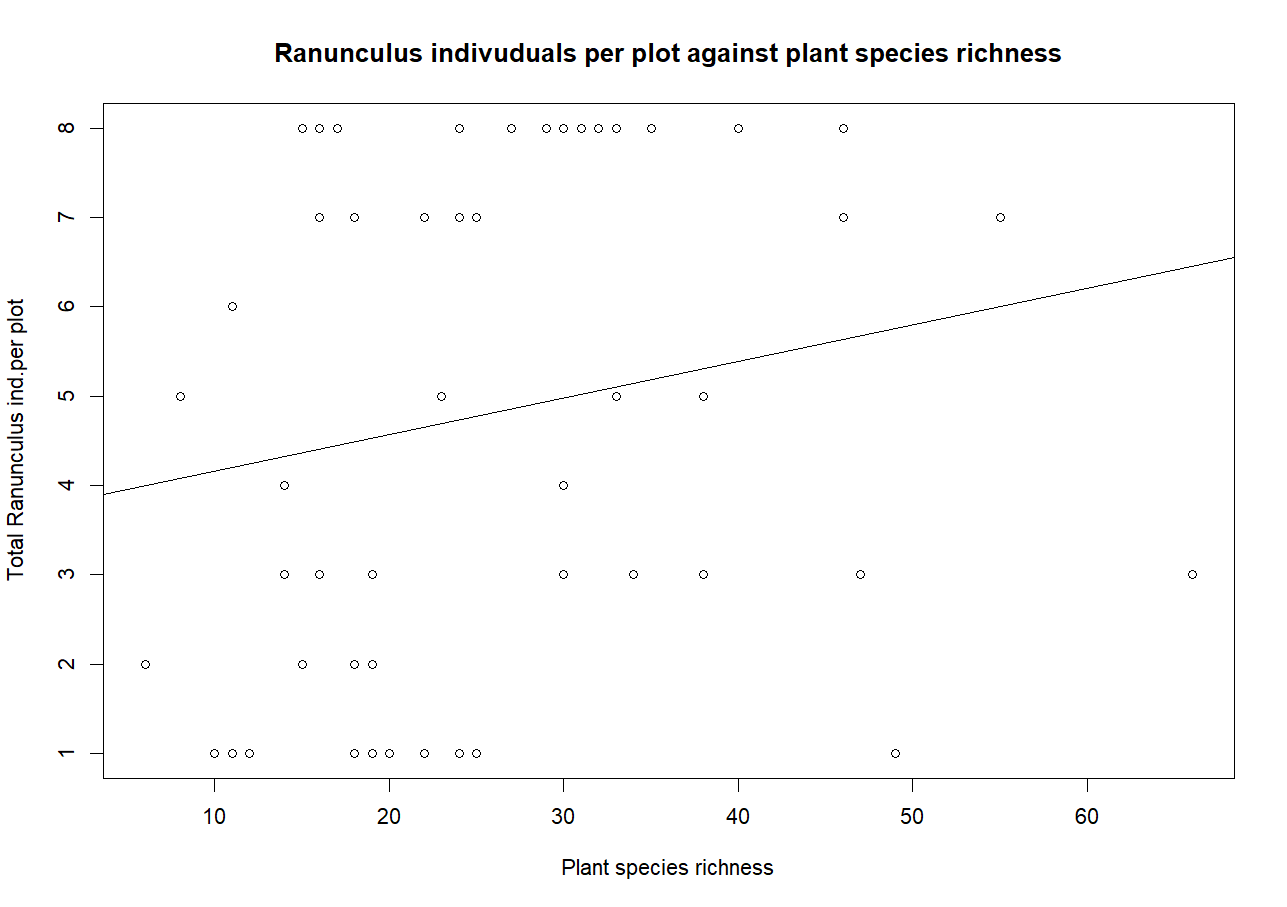
**

**Figure S11** Total number of Ranunculus individuals per plot in relation to surrounding plant species richness on grassland plots in Hainich-Dün and the Swabian Alb within the Biodiversity Exploratories. Pollinator data by Christiane Weiner from 2008 (BExIS dataset ID 15086, 4981, and 4963 (Weiner et al. 2019a; Weiner et al. 2019b; Weiner et al. 2022))

**
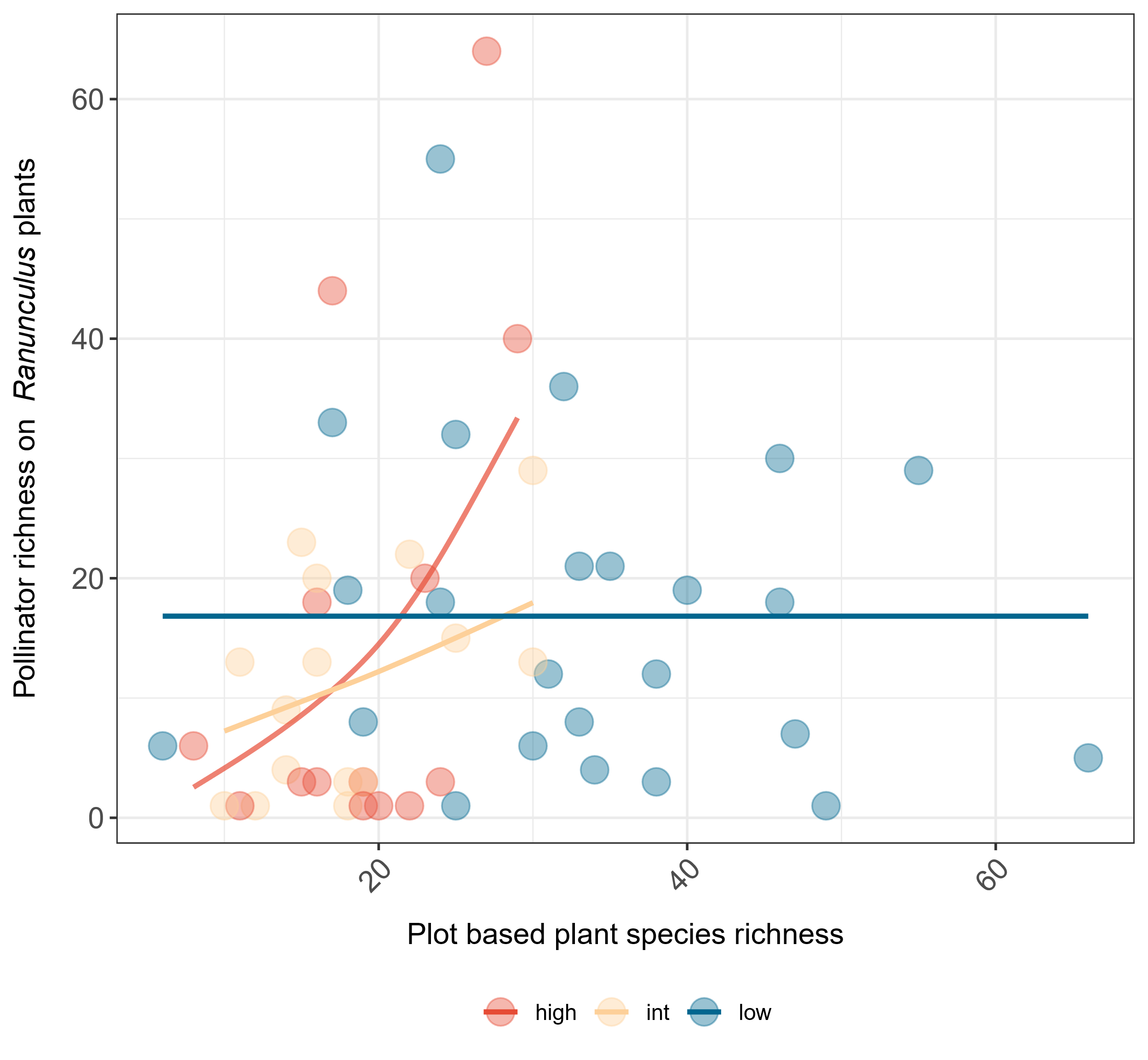
**

**Figure S12** Pollinator richness found on *Ranunculus* plants in relation to plant species richness on grassland plots differing in land-use intensity (LUI, color-coded: blue - low, yellow - intermediate and red – high) based on data from Weiner *et al.* (2014). Smoothed conditional means were added by using a geom_smooth, method = “gam” with the forrmula = 'y ~ s(x, bs = "cs").

**Table S5** Results for linear mixed-effects model testing for the effect of plant species richness at plots (Plant species richness) and categorized land-use intensity (LUI cat, categorized into high, intermediate and low land-use intensity) on pollinator richness on Ranunculus plants, with the total number of Ranunculus individuals per plot as a random factor (Total Ranunculus Ind); shown are Degrees of freedom (Df), Sum of Squares (Sum of Sq), Mean squares (Mean Sq), F-vaue and p-value (Pr>F) with significance (Sign.) codes: *** 0,001; ** 0,01; * 0,05; ns >0.05. Marginal R-squared value and conditional R-squared value were calculated using the MuMin package.

| **Richness pollinators on Ranunculus** | **DF** | **Sum Sq** | **Mean Sq** | **F-value** | **p-value** | **Sign.** |
| --- | --- | --- | --- | --- | --- | --- |
| *Plant species richness* | 1 | 284.89 | 284.89 | 4.4833 | 0.03941 | * |
| *LUI cat* | 2 | 366.08 | 183.04 | 2.8805 | 0.06584 | . |
| *Plant species richness* x *LUI cat* | 2 | 632.9 | 316.46 | 4.9801 | 0.01082 | * |

R²m: 0.12; R²c: 0.63

Additionally, we tested the richness and Shannon diversity of pollinators found on all flowering plant species ( *Richness/Shannon pollinators total plants*) for co-correlation using the same methodology as described above for *Richness pollinators on Ranunculus*. Both variables significantly correlated with each other (Figure S13), thus only Richness pollinators total was used in the linear model testing for the effect of *Plant species richness* on *Richness pollinators total plants*. Pollinator richness on flowering plants (*Richness pollinators on total plants*) correlated positively and significantly with *Plant Species Richness* (Figure S14, Table S5). LUI category (*LUI cat*) as well as the interaction of *LUI cat* and *Plant species richness* were significant.

**
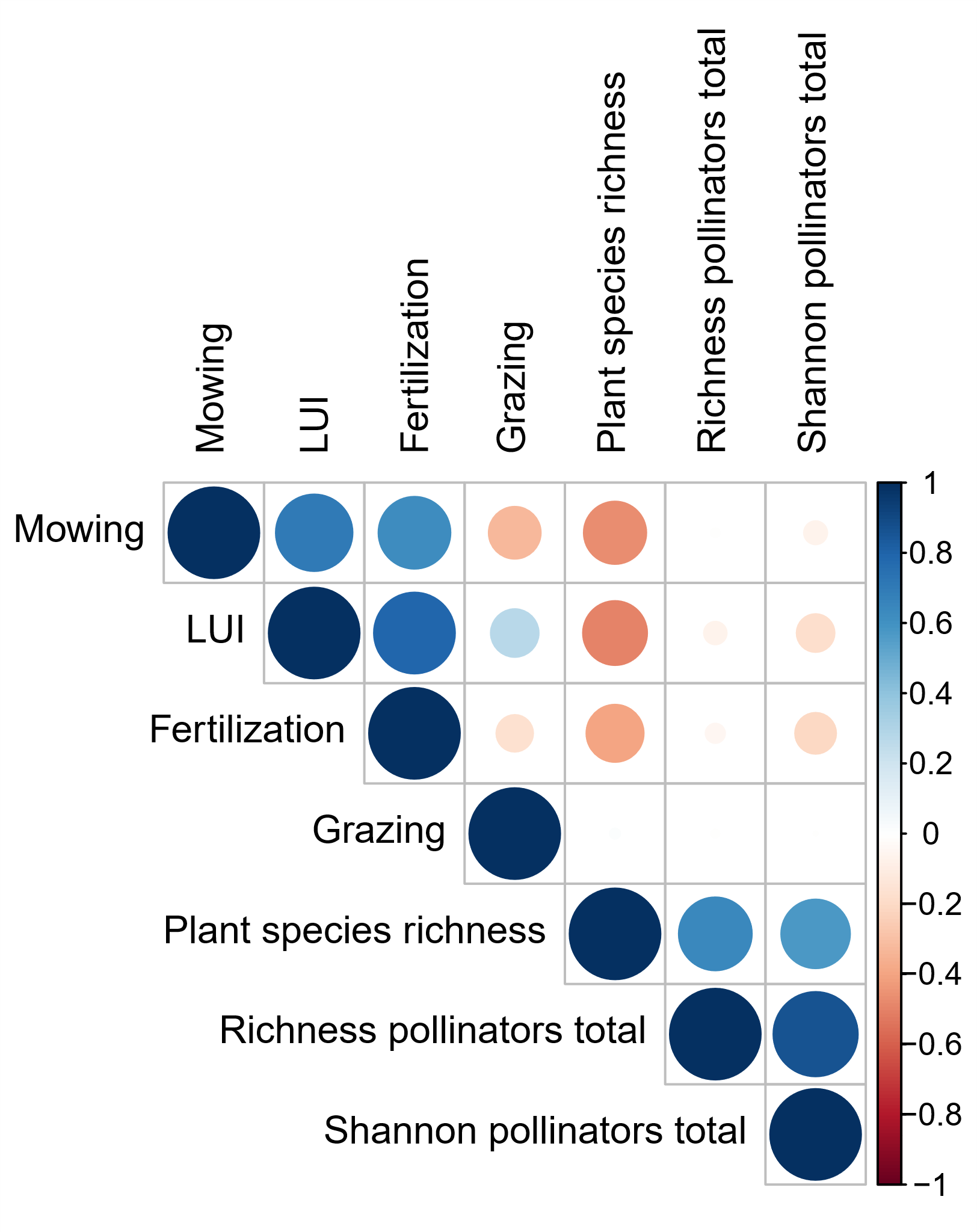
**

**Figure S13** Correlation matrix of response and explanatory variables for data from Christane Weiner 2008, including *Richness pollinators total plants* and *Plant species richness*, with a heatmap-like visualisation of the correlation coefficients where the variables are ordered based on hierarchical clustering.

**
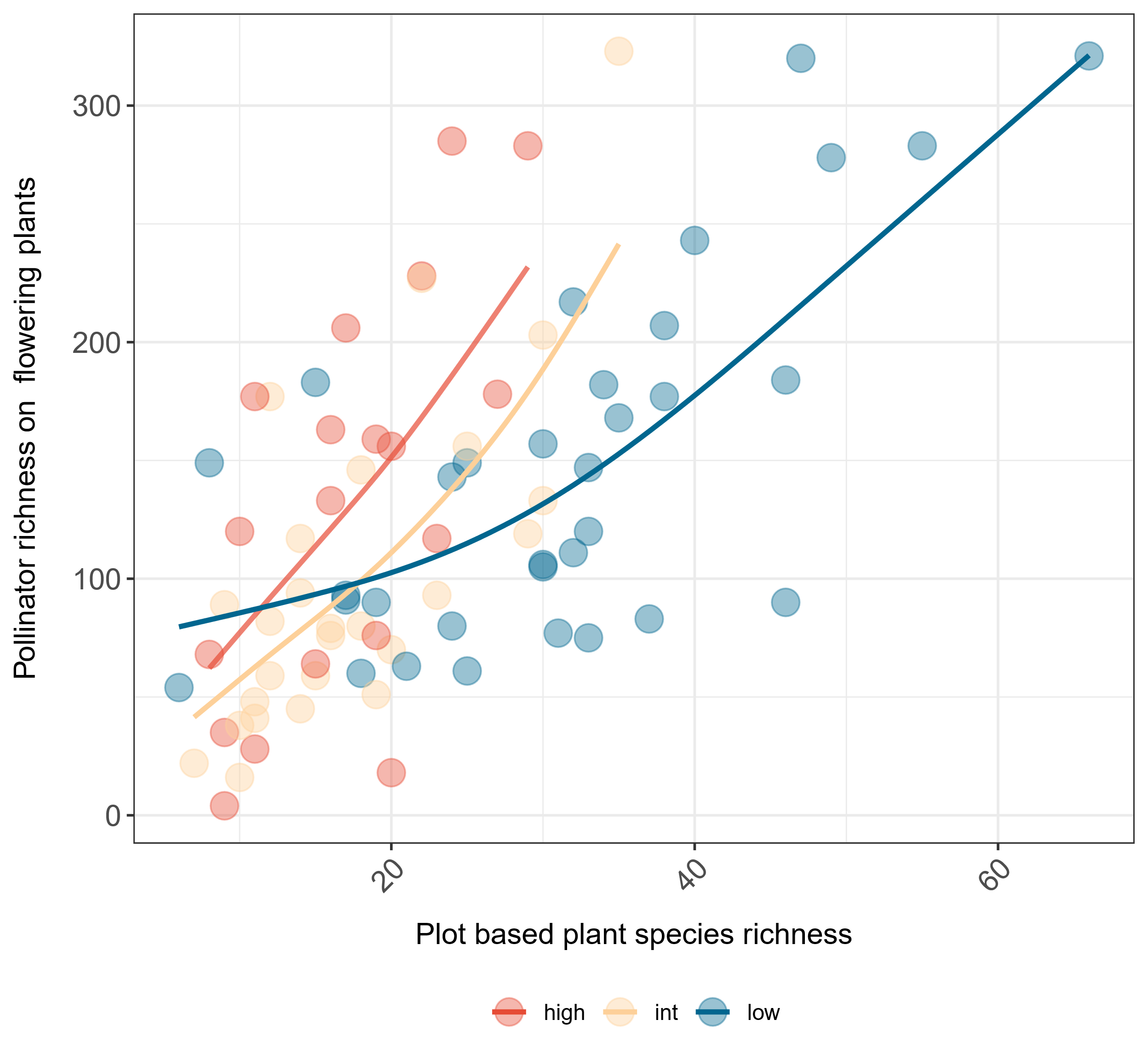
**

**Figure S14** Pollinator richness found on all flowering plants in relation to plant species richness on grassland plots differing in land-use intensity (LUI, color-coded: blue - low, yellow - intermediate and red – high) based on data from Weiner *et al.* (2014). Smoothed conditional means were added by using a geom_smooth, method = “gam” with the forrmula = 'y ~ s(x, bs = "cs").

**Table S6** Type III Analysis of Variance table with Satterthwaite’s Method for the effect of plant species richness at plots (Plant species richness) and categorized land-use intensity (LUI cat, categorized into high, intermediate and low land-use intensity) on pollinator richness on flowering plants (Richness pollinators on total plants). Shown are Degrees of freedom (Df), Sum of Squares (Sum of Sq), Mean squares (Mean Sq), F-vaue and p-value (Pr>F) with significance (Sign.) codes: *** 0,001; ** 0,01; * 0,05; ns >0.05.

| **Richness pollinators on total plants** | **DF** | **Sum Sq** | **Mean Sq** | **F-value** | **p-value** | **Sign.** |
| --- | --- | --- | --- | --- | --- | --- |
| *Plant species richness* | 1 | 194958 | 194958 | 62.0159 | 2.623e^-11^ | *** |
| *LUI cat* | 2 | 28657 | 14328 | 4.5578 | 0.01368 | * |
| *Plant species richness* x *LUI cat* | 2 | 20378 | 10189 | 3.2411 | 0.04490 | * |

Multiple R-squared: 0.519, Adjusted R-squared: 0.485

**Supplementary material – part 3: Observation data from 2020 and 2021 for wild bee species found on *Ranunculus* flowers**

For the EU-project NutriB2, observation data has been collected within the Biodiversity Exploratories on similar plots in the same regions as the stigma samples of this study (Parreño et al. 2025, publication in press, dataset ID: 31131). To further infer our results in a more ecologically meaningful way, we added this data, which is however only restricted to wild bees. Nonetheless, we think this data subset will share some more insight into the importance to survey heterospecific pollen on flowers, inferring patterns of pollination from the plant’s perspective. We filtered the dataset for bee species, which were detected on *Ranunculus* flowers via observation and calculated bee species richness per plot on *Ranunculus* plants (*Bee richness on Ranunculus*). We plotted *Bee richness on Ranunculus* against *Average plant species richness* (Data taken from Parreño et al. (2025), publication in press, dataset ID: 31131) and tested this relationship using a linear model, showing a significant effect of *Average plant species richness* on *Bee richness on Ranunculus* (Table S7, p = 0.0026). We also added LUI cat as an interaction term, but this was non-significant.

**
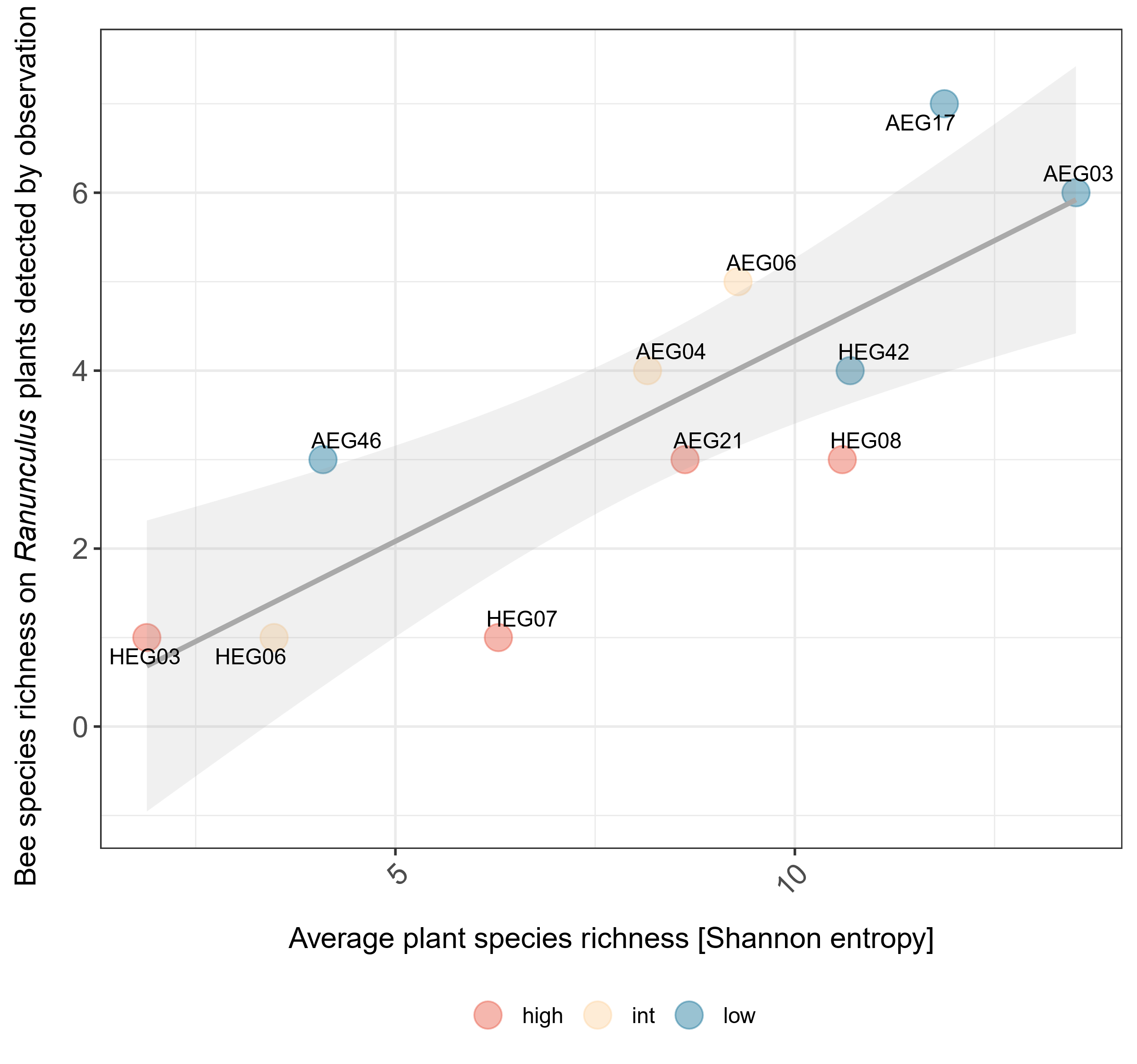
**

**Figure S15** Bee species richness found on *Ranunculus* plants (*Bee richness on Ranunculus*) plotted against *Average plant species richness*. Data was collected within the project NutriB^2^ in 2020 and 2021 (Parreño et al. (2025), publication in press, dataset ID: 31131 ), Plot differences based on land-use intensity (LUI) category are indicated by colour: (blue - low, yellow – intermediate, and red – high).

**Table S7** Type III Analysis of Variance table with Satterthwaite’s Method for the effect of Average plant species richness and categorized land-use intensity (LUI cat, categorized into high, intermediate and low land-use intensity) on bee species richness on Ranunculus flowers (Bee richness on Ranunculus). Shown are Degrees of freedom (Df), Sum of Squares (Sum of Sq), Mean squares (Mean Sq), F-vaue and p-value (Pr>F) with significance (Sign.) codes: *** 0,001; ** 0,01; * 0,05; ns >0.05.

| **Bee richness on *Ranunculus*** | **DF** | **Sum Sq** | **Mean Sq** | **F-value** | **p-value** | **Sign.** |
| --- | --- | --- | --- | --- | --- | --- |
| *Average plant species richness* | 1 | 27.901 | 27.901 | 30.737 | 0.0026 | ** |
| *LUI cat* | 2 | 6.033 | 3.323 | 4.5578 | 0.1208 | ns. |
| *Average plant species richness* x *LUI cat* | 2 | 2.255 | 1.128 | 1.242 | 0.3648 | ns. |

Multiple R-squared: 0.889, Adjusted R-squared: 0.777

**Supplementary material – part 4: Statistical model development (Stigma data)**

All variables, tested for collinearity are displayed in Figure S16. We found LUI to be strongly correlated with grazing, slightly with fertilization, and hardly with mowing. Mowing and fertilization were negatively correlated with plant species richness on plots “*Plant species richness”*. *Plant species richness* in turn correlated negatively with the Shannon diversity “*Shannon stigma*” and richness “*Richness stigma*” of pollen on *Ranunculus* stigmas. *Plant species richness* and *Plant species diversity* also highly correlated.


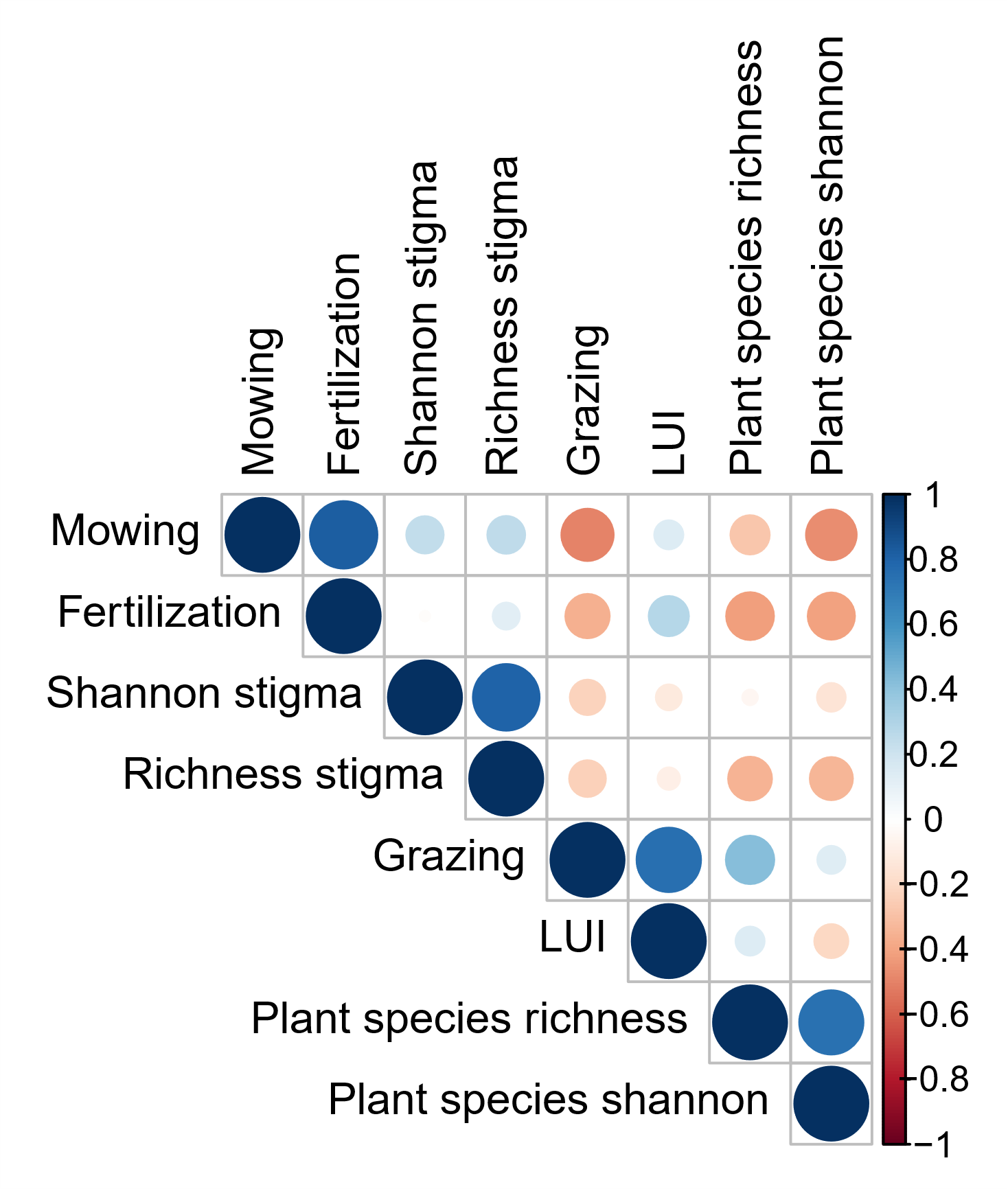


**Figure S16** Correlation matrix of response and explanatory variables for stigma metabarcoding data with a heatmap-like visualisation of the correlation coefficients where the variables are ordered based on hierarchical clustering.

We tested three different models to check if the plant species richness found on *Ranunculus* stigmas (*Richness stigma*), is affected by plant species richness on plots (*Plant Species Richness*) and in addition we also checked if the interaction between *Plant Species Richness* and *Ranunculus* species (*Host*) has an effect on *Richness stigma* (Table S8).

**Table S8** Comparison of different models testing the relationship between plant species richness found on stigma samples (Richness stigma), Ranunculus species (Host) and plant species richness on plots (Plant species richness) with corresponding Residual Sum of Squares (RSS) values and parameters returned by comparing models with Likelihood ratio tests (L.test). **M1: “Richness stigma ~ Plant species richness”; M2: “Richness stigma ~ Plant species richness + Host”;** **M3: “Richness stigma ~ Plant species richness² + Plant species richness * Host”.** Residual degrees of freedom (Res.Df), RSS (Residual Sum of Squares), DF (Difference in degrees of freedom between models), Sum of Squares (Sum of Sq), Mean squares (Mean Sq), F-vaue and p-value (Pr>F) with **s**ignificance (Sign.) codes: *** 0,001; ** 0,01; * 0,05; ns >0.05.

| **Model** | **Res.Df** | **RSS** | **DF** | **Sum of Sq** | **F-value** | **Pr(>F)** | **Sign.** | |
| --- | --- | --- | --- | --- | --- | --- | --- | --- |
| **M3** | 70 | 314.77 |  |  |  |  |  |  |
| **M2** | 72 | 336.49 | -2 | -21.716 | 2.4146 | 0.09681 | ns |  |
| **M1** | 74 | 357.93 | -2 | -21.444 | 2.3844 | 0.09959 | ns |  |

Model M3 has the lowest RSS value and has thus the best fit. We tested the model fit using the effects package (Smart *et al.* 2017) by creating affect plots, once for model M3 without a quadratic effect for *Plant Species Richness* (Figure S17 A) and once with a quadratic effect for *Plant Species Richness* (Figure S17 B)*.* This revealed that the model was best fitted assuming a quadratic effect of the independent variable *Plant species richness*. Model M3 has the lowest RSS value and has thus the best fit. We tested the model fit using the effects package (Smart *et al.* 2017) by creating affect plots, once for model M3 without a quadratic effect for *Plant Species Richness* (Figure S17 A) and once with a quadratic effect for *Plant Species Richness* (Figure S17 B)*.* This revealed that the model was best fitted assuming a quadratic effect of the independent variable *Plant species richness*.

**
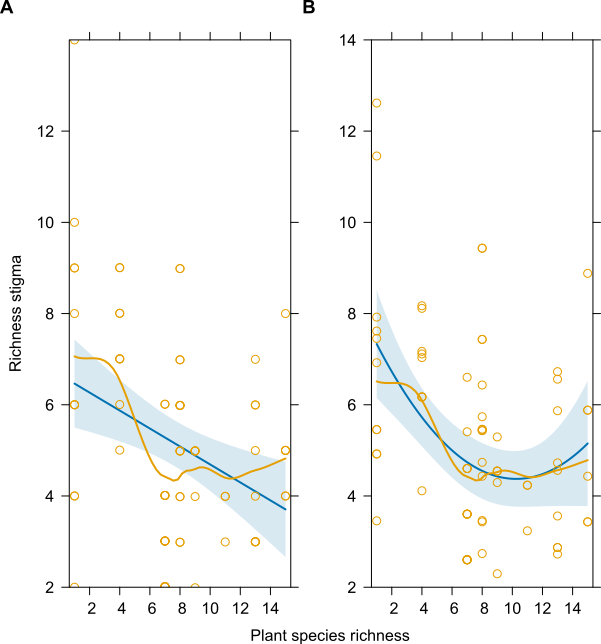
**

**Figure S17** *Plant species richness* effect plot for the response variable *Richness stigma* and the explanatory variable *Plant species richness* based on A) Model M3 (Table S7) without a quadratic effect, showing a misfit of the model and B) Model M3 with an added quadratic effect for *Plant species richness*. Plots were created using the effects package (Smart *et al.* 2017).

In addition, we tested whether Exploratory needed to be added as a random factor to the final model M3 including the quadratic effect.

*Test for host nested in plot nested in exploratory.*

**Table S9** Model comparison to test host nested in plot nested in exploratory for the established model M3. Significance codes: *** 0,001; ** 0,01; * 0,05; ns >0.05.

| **Model comparison** | **M1**  **lm** | **M2**  **lmer** | **M3**  **lmer** | **M4**  **lmer** |
| --- | --- | --- | --- | --- |
| **Response variable** | Richness stigma | Richness stigma | Richness stigma | Richness stigma |
| **Explanatory variable 1** | (Plant species richness)^2^ | (Plant species richness)^2^ | (Plant species richness)^2^ | (Plant species richness)^2^ |
| **Explanatory variable 2** | Host*Plant species richness | Host*Plant species richness | Host*Plant species richness | Host*Plant species richness |
| **Random factor** |  | (1\|bioregion) | (1\|bioregion/plot) | (1\|bioregion/plot/Host) |
| **AIC** | 331.86 | 333.86 | 332.78 | 331.57 |
| **Pr(>Chisq)** |  | 1.000 | 0.07791 | 0.0734 |
| **Significance** |  | ns. | ns. | ns. |

**References**

Bates, Douglas; Mächler, Martin; Bolker, Ben; Walker, Steve (2015): Fitting Linear Mixed-Effects Models Using lme4. In: *J. Stat. Soft.* 67 (1). DOI: 10.18637/jss.v067.i01.

Jari Oksanen; Gavin L. Simpson; F. Guillaume Blanchet; Roeland Kindt; Pierre Legendre; Peter R. Minchin et al. (2022): vegan: Community Ecology Package. Online verfügbar unter https://CRAN.R-project.org/package=vegan.

McMurdie, Paul J.; Holmes, Susan (2013): phyloseq: an R package for reproducible interactive analysis and graphics of microbiome census data. In: *PLOS ONE* 8 (4), e61217. DOI: 10.1371/journal.pone.0061217.

Parreño, Alejandra; Werle, Susanne; Buydens, Louella; Spitz, Joshua; Härtl, Franz; Montoya, Jeremias et al. (2025): Data on Visitation records from wild bees and plants along a land use gradient in Germany and Belgium. In: *Data in Brief*.

Sickel, Wiebke; Ankenbrand, Markus J.; Grimmer, Gudrun; Förster, Frank; Steffan-Dewenter, Ingolf; Keller, Alexander (2016): P. 3 Standard method for identification of bee pollen mixtures through meta-barcoding. In: *High-throughput biodiversity assessment‐Powers and limitations of meta-barcoding*, 26.

Smart, M. D.; Cornman, R. S.; Iwanowicz, D. D.; McDermott-Kubeczko, M.; Pettis, J. S.; Spivak, M. S.; Otto, C. R. V. (2017): A Comparison of Honey Bee-Collected Pollen From Working Agricultural Lands Using Light Microscopy and ITS Metabarcoding. In: *Environmental entomology* 46 (1), S. 38–49. DOI: 10.1093/ee/nvw159.

Weiner, Christiane; Linsenmair, Karl Eduard; Blüthgen, Nico (2019a): Flower availability 2008 Alb-korrigiert: Biodiversity Exploratories Information System. Online verfügbar unter https://www.bexis.uni-jena.de/ddm/data/Showdata/4981?version=2.

Weiner, Christiane; Linsenmair, Karl Eduard; Blüthgen, Nico (2019b): Flower availability 2008 Hainich: Biodiversity Exploratories Information System. Online verfügbar unter https://www.bexis.uni-jena.de/ddm/data/Showdata/4963?version=2.

Weiner, Christiane; Werner, Michael; Blüthgen, Nico (2022): flower visitor interactions 2008-only pollinators: Biodiversity Exploratories Information System. Online verfügbar unter https://www.bexis.uni-jena.de/ddm/data/Showdata/15086?version=3.

Weiner, Christiane Natalie; Werner, Michael; Linsenmair, Karl Eduard; Blüthgen, Nico (2014): Land-use impacts on plant-pollinator networks: interaction strength and specialization predict pollinator declines. In: *Ecology* 95 (2), S. 466–474. DOI: 10.1890/13-0436.1.

Wickham, Hadley (2016): ggplot2: Elegant graphics for data analysis. Second edition. Switzerland: Springer (Use R!).
